# Functionality and Morphology: Identifying *Si* Agricultural Tools from Among Hemudu Scapular Implements in Eastern China

**DOI:** 10.1007/s10816-015-9271-x

**Published:** 2016-01-06

**Authors:** Liye Xie, Xuejiao Lu, Guoping Sun, Weijin Huang

**Affiliations:** 1 University of Toronto, Mississauga, ON L5L1C6 Canada; 2 Hemudu Museum, Yuyao County, Ningbo City, Zhejiang 315414 China; 3 Zhejiang Provincial Institute of Cultural Heritage and Archaeology, Hangzhou City, Zhejiang 310014 China

**Keywords:** Agricultural tool, Hemudu culture, Rice cultivation, Neolithic China, Use-wear analysis, Experimental archaeology

## Abstract

Most Chinese archaeologists assume that the scapular implements used in the Hemudu culture in eastern China (7000–5000 BP) were the *si* agricultural implements (tools for breaking ground and turning soils over to assist in seeding) recorded in ancient Chinese literatures and, accordingly, assume the Hemudu culture was a farming society. However, ethnographic and historical literatures worldwide have suggested inconclusive functions for similar implements. We conducted a range of experiments under realistic conditions, including hide and plant processing and earth-working, followed by use-wear analysis, to identify the functions of the Hemudu scapular implements. The results suggest that no more than half of the implements were employed as *si* and that their penetrability and durability were rather limited. These findings help explain why Hemudu should not be labeled as a farming society. Through experimentation and use-wear analysis, we produced relatively large datasets that make a significant contribution to the identification of soil-derived wear patterns on bone tools. We also included quantitative measurements of soil properties to ensure similarities in use contexts between our experimental and archaeological analogies in order to reach reliable functional identifications. Our approaches and results, therefore, provided a solid base for re-evaluating previous research as well as building a standardized database of scientific value for future evaluation and adjustment, even if that future research is done in isolation and in different soil contexts.

## Introduction

**Fig. 1:**
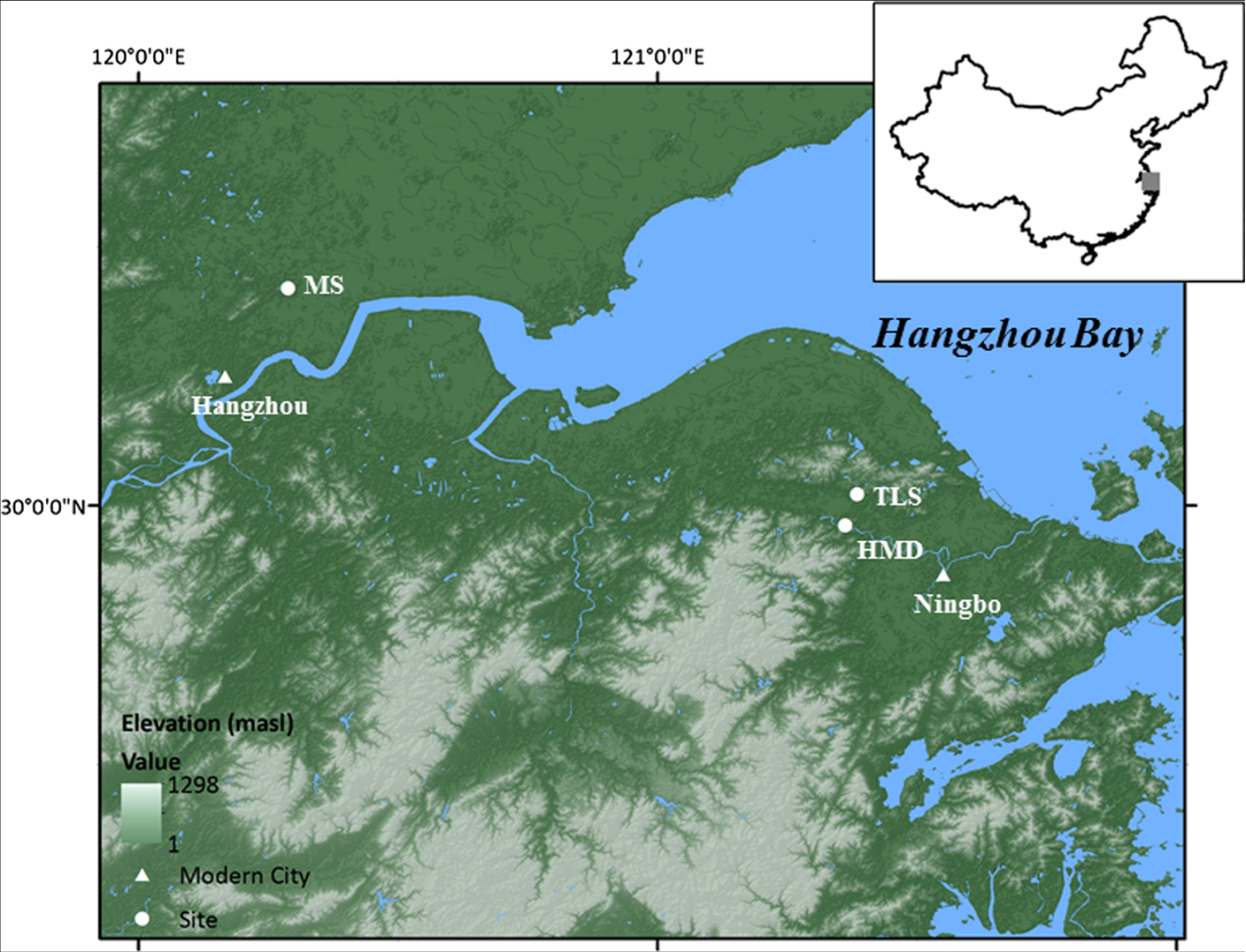
Map of the study area showing the sites of Hemudu (*HMD*) and Tianluoshan (*TLS*) in the Ningbo City, the two major sites of the Hemudu culture, as well as Maoshan (*MS*) in the Hangzhou City, a non-Hemudu culture site where we conducted some of our earth-working experiments

First discovered in the early 1970s, the Hemudu culture, dating to approximately 5000–7000 BP (Sun 2013; Wu et al. 2011; ZPICHA 2003) in the Lower Yangzi Basin (Fig. 1), is the Chinese archaeological culture best-known outside of China because its waterlogged condition has yielded well-preserved organic remains. Hemudu had been regarded as the earliest farming society in East Asia until the early 1990s, when older rice remains were found in the middle Yangzi Valley (Crawford and Shen 1998). Ironically, however, the Hemudu culture was not the subject of much scientific investigation until the last 10 years, when new archaeological findings from the Lower Yangzi Basin again drew attention to this area as the center of the origin of rice agriculture. Results from recent research show that thousands of years separated the appearance of incipient rice cultivation and the well-established agricultural societies in the Lower Yangzi Basin (Fuller et al. 2007; Liu et al. 2007; Pan 2011; Zheng et al. 2012).

Hemudu represents one of those societies in which rice cultivation was practiced over millennia, yet full-scale agriculture did not develop there spontaneously. Previous research on the process of agricultural development has focused on a combination of ecological and economic perspectives (*e.g.*, climate, environment, biological adaptations; see Fuller et al. 2009; Purugganan and Fuller 2009; Zheng et al. 2012). Our research focuses on agricultural implements, a perspective that has been largely neglected, to understand the prolonged process of the development of plant agriculture in the Hemudu culture. Farming implements are an indispensable component in agricultural practices. In the Hemudu culture, wooden digging sticks have been found in small numbers; spade-like implements were more common, mostly crafted from bone with a few made of medium-hard wood and stone (Xie 2014). The bone spades, crafted from large mammal scapulae, are frequently found at the sites of Hemudu and Tianluoshan, the only two sites of the Hemudu culture that have been systematically excavated. These bone spades are often uncovered together with artifacts and ecofacts for daily use and/or from daily refuse in the habitation area. Because these scapular implements are morphologically similar to descriptions of tilling or soil loosening tools in ancient Chinese text, or *si* (Chen 1980; Xu 1983), most archaeologists in China have labeled them as the *si* tools that represent an advanced Neolithic farming technology involving tillage (Chen 1980; You 1976). Together with the presence of abundant rice remains at the Hemudu site, this led researchers to assume the Hemudu culture was a farming society. New discoveries and excavations at Tianluoshan, a waterlogged site with 6.3 ha of rice fields lying west of a 3-ha village belonging to the Hemudu culture (Sun 2013; Zheng et al. 2009), have provided opportunities for systematic data collection and thorough research on Hemudu subsistence strategies.

Results of the most recent research have led to the conclusion that the Hemudu communities consumed a broad-spectrum diet consistent with low-level food production (Pan 2011; Qin et al. 2006), thus inviting a scientific examination of the hypothesized *si* agriculture. In particular, we ask two questions. Were the scapular implements *si*? If so, how effective were they? This paper focuses mainly on the first question; detailed discussion regarding the second question can be found in another paper (Xie et al. 2015).

To determine whether the scapular implements were *si*, we examined previous literature on similar implements, conducted experiments to test the major functions previously suggested for scapular implements, and then compared the use-wear patterns and morphological details of the experimental samples to the archaeological specimens.

## Methods

The pre-industrial use of scapular tools is a global phenomenon. In addition to the Lower Yangzi Basin in eastern China (ZPICHA 2003; ZPICHA and XM 2004), scapular implements have been found in Neolithic and early Iron Age contexts in many world regions, including the Upper and Middle Yellow River Valleys in north China (Andersson 1925, p. 15; SPIA and BMAT 2006), southern England, southern Germany, Swiss lake dwellings (Curwen 1926; Steppan 2001), and the Northern Plains of the USA (Griffitts 2006). Scapular implements were used until the early twentieth century by societies including various Native American groups (Bradbury and Bywater 1817, p. 175; Catlin 1844; Forde 1934; Wilson 1921), the aboriginal inhabitants of Sakhalin (Pilsudski and Majewicz 1992), and ethnic minorities in China such as the Jingpo (Kachin) of Yunnan (Song 1979; Wang 1991; Yin 1996).

Previous research has taken into account evidence from linguistic research, historical and ethnographic examinations, and brief experimentation. The results suggest diverse but uncertain functions for scapular implements. For example, Curwen (1926) pointed out that the term “shovel” and/or “digging” overlapped with the term “scapulae” at some period in Europe, indicating that scapulae might have once been commonly used as shovels. Photographic records in ethnographic accounts in southwestern China (Yin 1996) and pictorial stones from the Han Dynasties (206 BCE to 220 CE) in China also depict the use of morphologically similar implements in a shovel^1^ manner (Birrell 1993, p. 48). In contrast, ethnographic literature from North America depicts the use of scapulae as hoes^2^ (Wilson 1917, 1934). However, ethnographic records also suggest that scapular tools are too fragile to penetrate earth (Wang 1991; Wilson 1917). Experimental tests of functionality also led to inconclusive results. For example, after digging with shovels and hoes crafted from cow scapulae, Davis (1965) concluded that these implements are effective, while Evans and Limbrey (1974, pp. 199–200) reached an opposite conclusion with unmodified ox and horse scapulae.

Additional functions suggested for scapular implements include (1) processing bark (*e.g.*, Barrett 1933; Hoffman 1896, pp. 260–267; Skinner 1921); (2) softening and dressing hides (*e.g.*, Bell 1971; Grinnell 1962, p. 216; Hofman 1980); (3) stretching thongs (Campana 1989, p. 108); (4) wrenching, straightening, and polishing shafts (Campana 1989, p. 108); and (5) cutting weeds and dry grass, moving wood chips, clearing snow, cleaning fireplaces and animal pens, and arranging already loosen soil in the field (Wang 1991; Wilson 1934). Use-wear analyses on archaeological samples from North America also point to multiple functions for the scapular implements (Griffitts 2006).

Apparently scapular implements could have had multiple functions. However, none of the functional identifications mentioned above can be accepted without caution. The diverse functional interpretations of the scapular implements reflect problems associated with the direct use of ethnographic and historical evidence for functional interpretations of archaeological samples. Preliminary use-wear analysis and a small number of experiments conducted by Griffitts (2006), Davis (1965), and Evans and Limbrey (1974) do not adequately clarify the puzzles suggested in different reference resources regarding the implements’ functions.

The morphologies of scapular implements and their use contexts vary across culture, area, and period. Therefore, one should not rely without caution on historical, ethnographic, or experimental data for cross-cultural and/or cross-regional comparison. However, it can be very useful to be able to identify universal principles beyond the limit of culture and region when studying global phenomena in material culture. Therefore, our research was designed with two goals: (1) to identify whether *si* tools are among the Hemudu scapular implements and (2) to provide data that can be useful beyond the Hemudu case that motivated the research.

The first author closely examined the morphologies of the archaeological scapular implements and used them as models for replicas. She then designed a set of experiments with reference to ethnographic accounts from various world regions to test the major functions previously suggested for scapular implements and to obtain use-wear patterns. Given that soil-derived wear is underrepresented in prior research (compared to hide- and plant-derived wear), our experiments focused mostly on this task. With a set of controlled experiments under realistic conditions, in a range of representative soils with which the prehistoric farmers in the Lower Yangzi Basin would have had to contend, criteria were developed for identifying soil-derived wear on bone tools, along with quantitative descriptions of soil properties in the experimental fields. This allows (1) us to identify bone earth-working implements and their use contexts in the Hemudu culture and (2) future researchers to evaluate and/or adjust our experimental results according to similarities in soil substrates when applying our data to other areas.

## Raw Materials and Morphologies of the Hemudu Scapular Implements

The majority of scapular tools found, both ethnographically and/or archaeologically, in the Northern and Central Plains of North America are made from bison and elk scapulae (Griffitts 2006, p. 272). In the ethnographic literatures worldwide, the cutting edges of the scapular implements recorded as hoes or shovels were relatively straight and sharp (Wilson 1917: Fig. 2; Yin 1996: Fig. 138). In contrast, the morphology common to scapular tools regarded as bark and/or hide processors is that the inner parts of the blades were either perforated or cut away to form a two-pronged edge (Hofman 1980: Fig. 1).

The hafting modifications of the scapular implements from European and American prehistoric and historic societies are relatively simple. In many cases, the modifications only include the removal of the scapular spine and the posterior border of the bone and slight modification on the sides of the necks (Griffitts 2006: Figs. 5.4 and 8.1; Lehmer 1971: Fig. 55; Steppan 2001: Fig. 4). Some samples have a hole at the center of the glenoid cavity or an opening on the ventral surface of the bone (Steppan 2001, pp. 2–3, 6–9; Lehmer 1971: Fig. 55).

In our research, we studied all accessible scapular tools recovered from the sites of Hemudu and Tianluoshan, the waterlogged sites where most Hemudu scapular implements have been discovered. Examined samples included (1) 93 scapular tools housed at the Hemudu Museum, representing about half of all scapular tools collected from that site in the 1970s, and (2) 62 scapular tools housed at the Tianluoshan Archaeological Station, representing 95 % of scapular tools excavated from the Tianluoshan site between 2004 and December 2011. The majority of these implements were crafted from the scapulae of Cervidae and Bovidae. Occasionally, scapulae sourced from members of the Ursidae were also used. With reference to the local archaeological fauna (Wei et al. 1990; Zhang et al. 2011), and for simplicity’s sake, we use the common names of these animals, deer, water buffalo, and bear, in our discussion. Details of source animals for all examined scapular tools are listed in Table 1.

**Table 1:** Numbers of examined samples

Location	Tool	Water buffalo	Deer	Bear	Undet.
HMD Museum	93	63	20	2	8
TLS	62	15	16	0	31

The “undet” (undetermined) source animals are still mostly deer and water buffalo, but they cannot be named as one or another because their diagnostic portions are missing

**Fig. 2:**
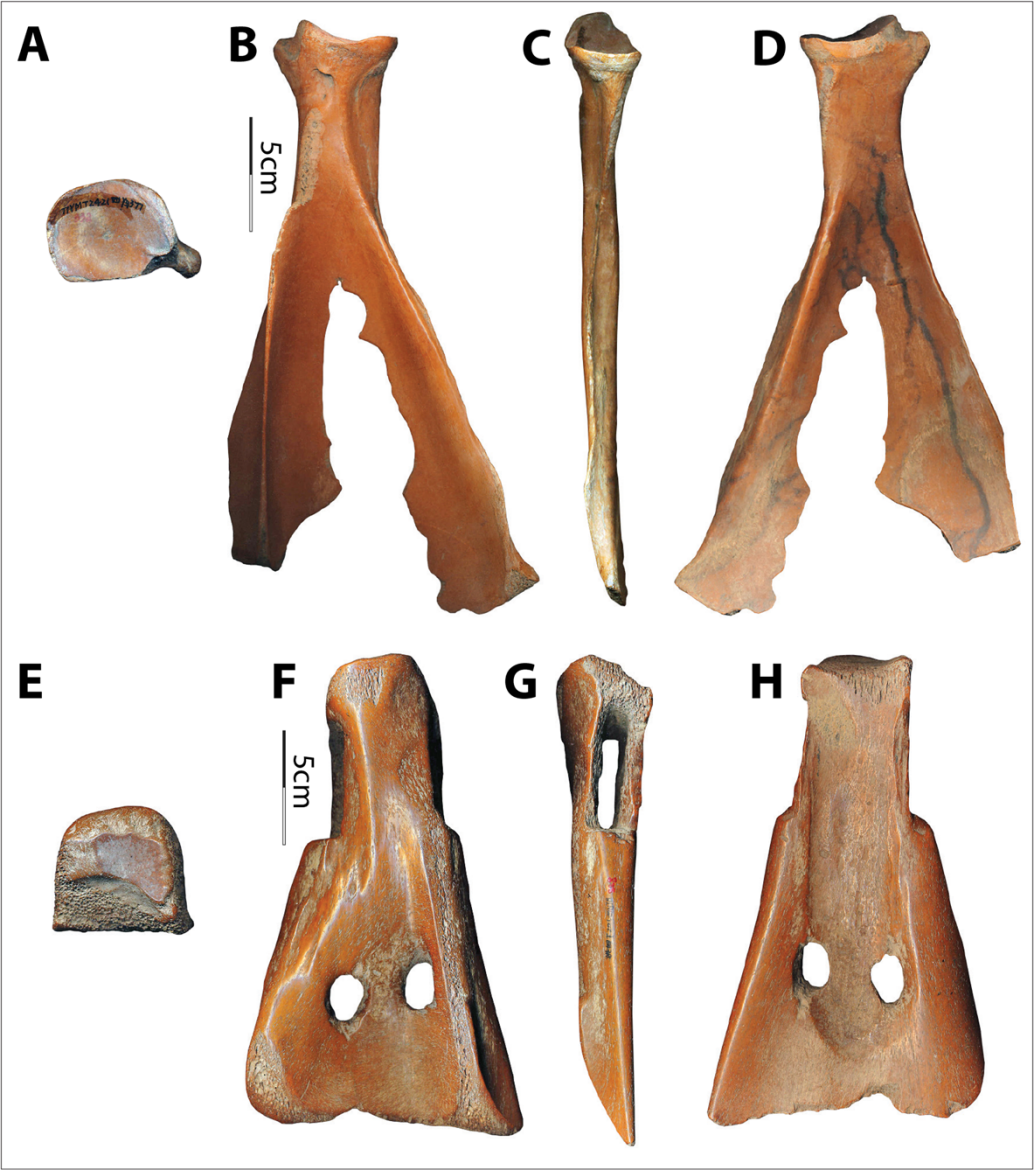
Two morphologies of scapular implements from the Hemudu culture. **a**–**d** Two-pronged or V-edged example (top, dorsal, lateral, and ventral views of HMD 332). **e**–**h** Straight-edged or E-edged example (top, dorsal, lateral, and ventral views of HMD 328)

**Fig. 3:**
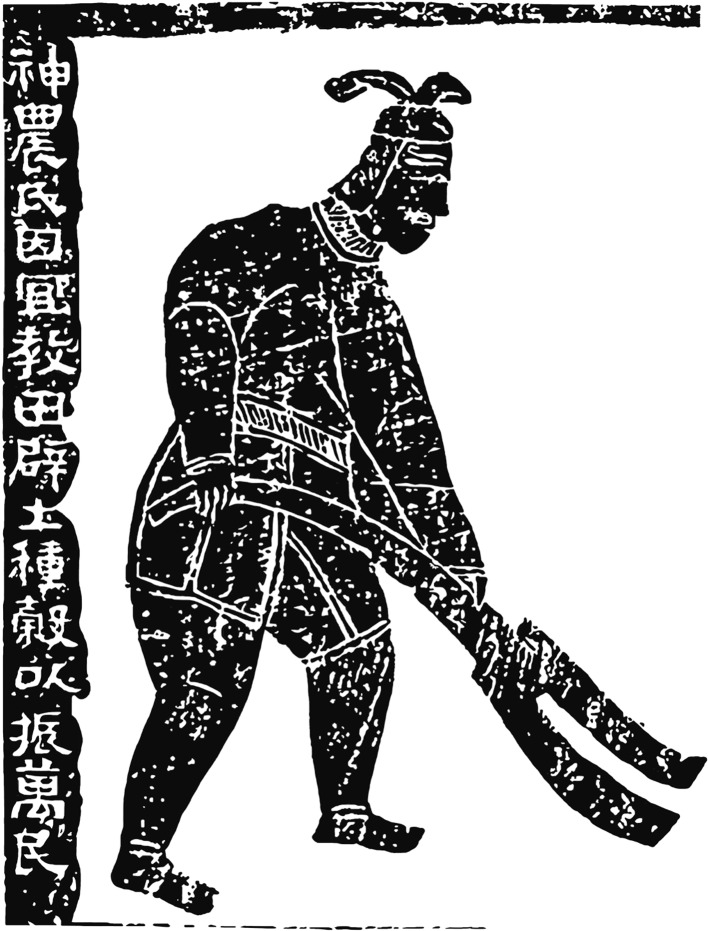
Carved image dating to AD 151 from the Eastern Han dynasty, on a pictorial stone at the Wuliang Shrine, depicting the Divine Farmer (Shen Nong) with a V-edged implement (after Li 1914, p. 1)

Both straight-edged (E-edged) and two-pronged (V-edged) scapular implements were used in the Hemudu culture (Fig. 2). Huang (1996) proposes that these different morphologies may have begun as the same edges but been abandoned at different stages of wear from penetrating earth. The confidence of Chinese archaeologists who assumed that the V-edged scapular tools were used as soil loosening implements came from morphologically similar implements found in pictorial stones from the Han Dynasties, 206 BCE to 220 CE. For example, one carved image depicts the Divine Farmer (Shen Nong) with a V-edged implement and the following inscription: “The Farmer God taught agriculture based on land use; he opened up the land and planted millet to encourage the myriad people” (神农氏因宜教田,辟土种谷,以振万民) (Fig. 3). Wooden implements of similar morphology, sometimes with metal “teeth,” are also frequently found in Chinese Iron Age tombs, mines, and pits, implying that such tools were employed in construction processes (Chen 1980; Wang 1995; Li 1987).

**Fig. 4:**
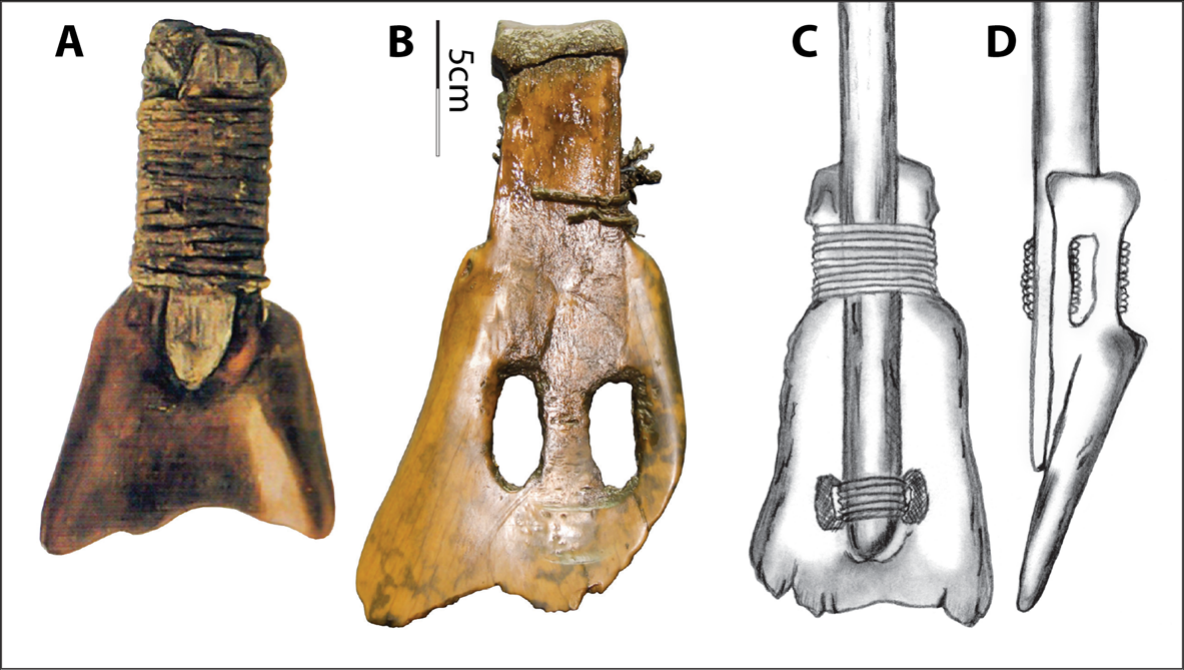
Hafting styles for scapular earth-working implements. **a** HMDT224(4)B:175, 18.2-cm long and 9.8-cm wide, with lashing materials and a small portion of the shaft (after ZPICHA 2003: plate 26–1). **b** TLST302(8):3 with lashing materials. **c**, **d** Reconstructed hafting method suggested by Mou (1980)

The Hemudu specimens include examples of extremely simple hafting modifications, with only slight removal of some of the scapular spines and very slight modifications on the sides of the necks (Fig. 2a–d). However, some Hemudu specimens show very sophisticated modifications for hafting, including a groove on the ventral face with two perforations and notches on the lateral sides of the scapular neck, which are usually transversely scored through (Fig. 2e–h). Two scapular implements were found with lashing materials and a small portion of a handle, suggesting that they were hafted as shovels and that the ligatures were lashed not only across the sockets but also completely around the necks, tightly fastening the shafts (Fig. 4a, b). Accordingly, researchers have reconstructed the hafting technique of the Hemudu-style scapular earth-working implements (Fig. 4c, d). We applied this hafting and fastening technique in our digging experiments.

## Previous Studies of Use-Wear Traces on Bone Tools

The most common experiments conducted by bone use-wear analysts involve processing animal and plant tissues. The patterns derived from working animal and plant fibers, shown in results from experiments conducted by different researchers, are relatively consistent (*e.g.*, Buc 2011; Griffitts 2006; Legrand 2008; LeMoine 1997). Table 2 summarizes use-wear signatures of plant and animal fibers reported in the extant literature. These use-wear patterns were replicable, and they are supported by comparative observations between experimental and ethnographic collections (Stone 2011) as well as fundamental tribological analyses (*e.g.*, LeMoine 1994; Stone 2011).

**Table 2:** Summary of use-wear signatures of plant and animal fibers reported in publications (modified from Stone 2011: Table 7.1, with reference to Buc and Loponte 2007, Buc 2011, Christidou and Legrand 2005, Griffitts 2006, Legrand and Sidéra 2007, Legrand 2008, LeMoine 1997, Maigrot 2008, and Stone 2011)

Worked material	Polish	Striations	Other markers
Plant	Non-invasive; planar (especially silica-rich plants)	Shallow; long; *fine or variable*; *parallel or variable*; size rarely varies	Smoothed microtopography
Animal	Invasive	Irregular; rounded shape; smoothed or polished base; crossing; short; *shallow or deep*; size may or may not vary	Pitting; rounded microtopography
General to soft materials			Rounded volume loss

Italics indicate disagreement on diagnostic patterns for different kinds of wear. The term invasive refers to wear extending from the high points of asperities to their edges and may even go down to the interstice

In contrast, bone wear derived from earth-working has rarely been explored experimentally and the results are not comparable across studies (Table 3). The only wear pattern repeatedly observed by different researchers consisted of striations perpendicular to the implement’s edge. Although several researchers acknowledge that soil conditions, such as composition and moisture content, significantly affect wear patterns on bone implements (*e.g.*, Griffitts 1993; Rabett 2005), prior to our research no systematic experiments and use-wear analyses specifically addressing this had been conducted.

**Table 3:** Summary of use-wear derived from soil on bone and antler reported in publications

Working material	Polish	Striations	Other markers	Used time	Reference	Sample size	Hafted modes
Bone		Adjacent to the edge; perpendicular or diagonal to the edge	Macroscopically visible ripple marks	Hours	Davis (1965), Semenov (1970)	2	Hoe and shovel
Bone		Perpendicular to the edge	Rarely produced terminal fractures		Rabett (2005)		Shovel?
Bone	Patched; planar; bright as well as generic weak	Deep, uneven, roughly parallel, perpendicular to the edge	Pitting; cracking; macroscopically visible undulating and rounded edges		Griffitts (1993, 2006)	5	Unhafted
Antler	Rough	Light, coarse, or absent		40 min	LeMoine 1997	3	Pick

## Our Experiments and Results

### Experimental Designs

Our experiments incorporated working with the three main materials—soil, hides, and plants—that had been suggested for scapular tool use in previous publications. Given that soil-derived wear is under-represented in previous research compared to hide- and plant-derived wear and that the use-wear signatures of soil are far from clear, our experiments focused mostly on this task. Our experiments included 21 bone tools, two for hide-processing, two for bark-processing, and 17 shovels or hoes used in 11 fields.

**Fig. 5:**
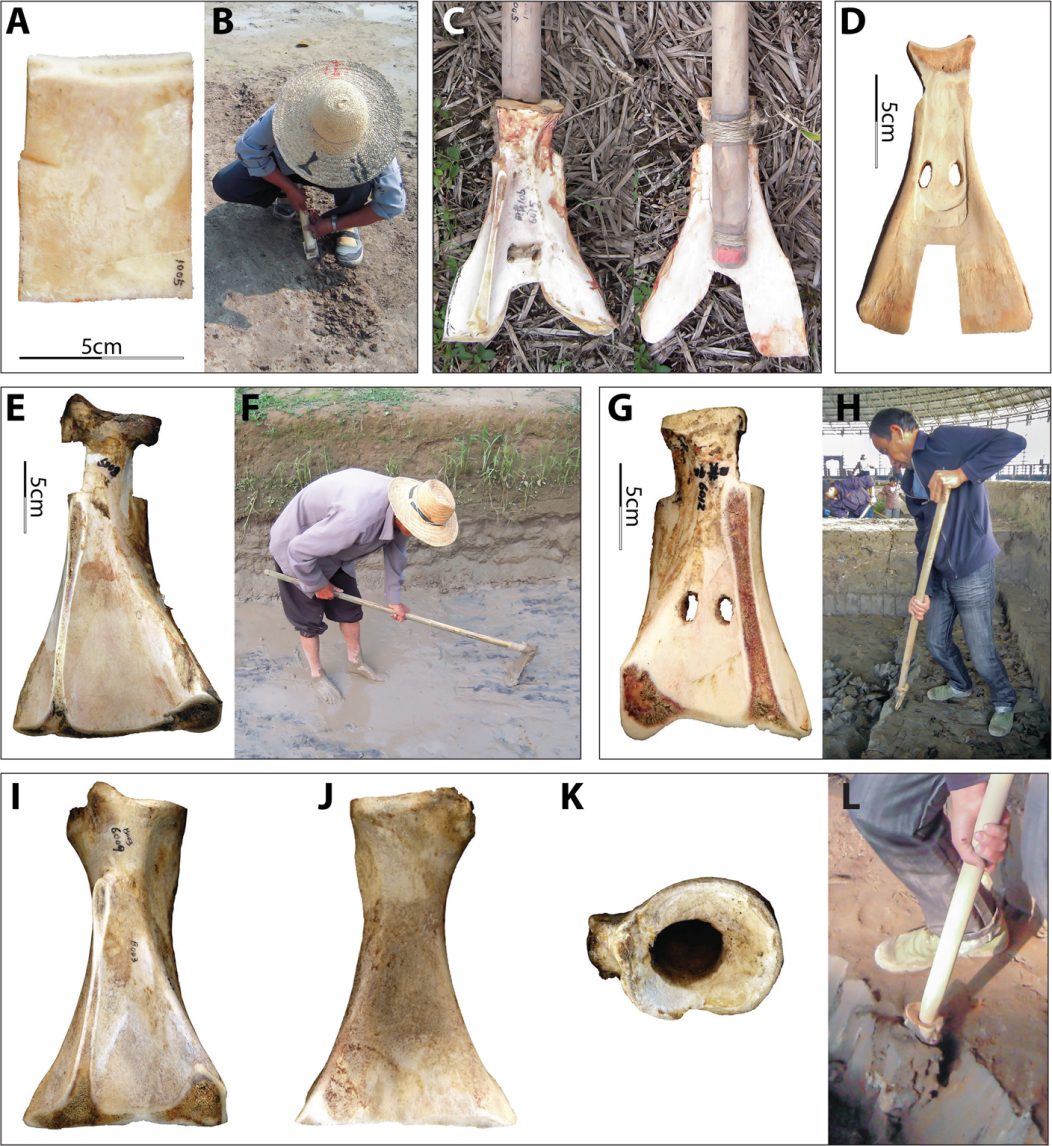
Examples of experimental specimens. **a**, **b** Dorsal view and use mode of no. 1005. **c** Dorsal and ventral views of no. 6015 with the handle on. **d** Ventral view of no. 6005. **e** and **f** Dorsal view and use mode of no. 6008. **g** and **h** Dorsal view and use mode of no. 6012. **i**–**l** Dorsal, ventral, and top views and use mode of no. 6009

Details of the experimental implements are listed in Table 4 with examples shown in Fig. 5. Most of our experimental implements were made from modern cattle scapulae collected from an abattoir in the Tianluoshan village and used to replicate full-size scapular tools (experimental implements with codes in the 6000 series in Table 4). Smaller bone blades (with codes in the 1000 series in Table 4) cut from the medial ends of cattle scapulae were used in a few cases due to a lack of supply of full-size replicated implements; the action performed was similar to that performed with a full-sized tool. The full-sized scapular tools included both V-edged and E-edged examples.

**Table 4:** Experimental implements

Tool no.	Worked materials	Edge morphology	Bone age^a^ (months)	Weight (g)	Length (cm)	Edge length^b^ (cm)	Edge angle^c^ (°)	Field no.	Hafted mode	Use time (min)
1004	Soil	E, slightly convex	3	20.4	7.53	4.29	18–20	XI	Shovel	90
1005	Soil	E, slightly concave	0.5	19.6	7.22	4.93	21–23	X	Shovel	90
6002	Soil, weed	E, slightly concave	11	368	20.8	15.4	30–35, A55	I and II	Shovel	30
6003	Soil, weed	E, slightly convex	11	417	26.1	19	26–30	II	Shovel	7
6004	Soil	E, slightly concave	3	325	21.2	13.03	30–33, A40	IX	Shovel	172
6004	Soil	E, slightly concave	12	311.5	20.3	13.13	30–35, A40	VI	Shovel	50
6004	Soil	E, slightly concave	12	311.3	20	13.1	25, A40	VII	Shovel	36
6005	Soil	EV	3	433	29.3	8.5	E edge: 25, S33; V edge: 55 and 45	IX	Shovel	138
6006	Soil	E	3	364	26.5	19.5	33, S40	IX	Shovel	85
6007	Soil	E	0	446	27.5	18.5	24–30	IX	Hoe	95
6008	Soil	E	0	377	22.5	15.5	26–32	IX	Hoe	20
6009	Soil	E, slightly concave	4	356	20.6	14	32–34	VIII	Shovel	83
6011	Soil	E, slightly concave	8	250	18.6	10.7	25–29	III	Shovel	8
6012	Soil	Concave	0.5	286	19	11.5	20–27	VIII	Shovel	102
6013	Soil	E, slightly concave	2	249	18.7	11.5	22, A30	II	Shovel	182
6014	Soil	E, slightly concave	0.5	398	24.2	15.3	18–23, A30	VIII	Shovel	84
6015	Soil, weed, rice stem	EV	0	423.4	25.42	7.4	E edge: 25–26; V edge: 59 and 42	II	Shovel	n/a
6020	Soil	E	9	417	23	15.5	30–37	IV	Shovel	26
6021	Soil	E, slightly concave	13	263	18	13	20–22, A40	V	Shovel	20
6001	Hide	V	0	300	16.4	8.7	n/a	n/a	Attached to a tree	60
6017	Hide	V	1	318	29	11.8	n/a	n/a	Shovel handle attached to a pole	26
6018	Bark	V	3	289	28	8.34	n/a	n/a	Attached to a tree	164
6019	Bark	V	4	151	24	8.3	n/a	n/a	Attached to a tree	300

Both the medial end and two prongs of the EV-edged specimens are uni-beveled, but the two prongs of the V-edged specimens do not have beveled edges

*E* straight edge at the medial end of the bone, *V* two-pronged, *EV* coexistence of E and V edges

^a^Bone age means how long the bone had been extracted from the animal before it was used

^b^The edge length for the V edge is the depth of the notch

^c^The angles of each E edge vary; the angles on the borders or the spines are usually larger than those of the rest of the edges. In the column of “edge angle,” pure numbers or number ranges indicate the angles of most of the edges, the numbers following an A are the angles on the anterior borders, and the numbers following an S are the angles on the spines

All of the E-edged examples are uni-beveled, except the anterior border of no. 6002, which was bi-beveled. The edges of mimic implements were all straight. Due to constraints imposed by the natural morphology of bovine scapulae, some of the straight edges of the full-sized replicas are slightly concave or convex and each edge has inconsistent angles. To evaluate the functional significance of the sophisticated Hemudu hafting modifications for earth-penetrating, one of the E-edged scapular implements was made in the non-Hemudu style—with a hole at the center of the glenoid cavity for hafting (no. 6009, see Fig. 5i–l)—and its performance characteristics were compared to those of the implements with sophisticated modifications for hafting.

Replicated scapular tools with two-pronged edges used for earth-penetrating had sharp edges along the ends as well as along the inner margins of the two prongs (listed as EV-edged implements in Table 4) while those used for hide- or bark-processing had no sharp edges (listed as V edge in Table 4), as these are not indicated in ethnographic and archaeological accounts of this sort of function.

The processed hides came from cattle and were collected within a few days of removal from the animal. Due to budget limitations, hide strips rather than large pieces were used. The experiments resembled hide-softening processes recorded by Hofman (1980). Bark-processing experiments resembled the process of fiber extraction to produce twine or cordage (*e.g.*, Barrett 1933; Hoffman 1896, pp. 260–267; Skinner 1921). Bark was removed from freshly cut paper mulberry (*Broussonetia papyrifera*) branches and soaked in water for 2 to 3 days before being processed.

The 17 experimental shovels and hoes were used in 11 fields, covering a range of soil types that the users of prehistoric scapular tools would have had to contend with in the Lower Yangzi Basin (Table 5). The textures of these fields resemble and/or are identical to those of the initial residential surface, rice fields, and anthropogenic deposits in the burial and habitation zones associated with Tianluoshan (a site of the Hemudu culture) and Maoshan (a non-Hemudu culture site). All of the ancient fields used in the experiments date to 7000–4000 BP, spanning the periods of scapular tool use as well as subsequent periods in the Lower Yangzi Basin (see Xie et al. 2015 for additional descriptions of the cultural contexts of the sites). Because we (the researchers) lacked relevant experience, we hired 33 local farmers and laborers who grew up using hand farming tools to conduct the experiments.

**Table 5:** Cultural contexts and soil physical properties in the experimental fields with associated experimental implements

Field no.	Context	Location	Date (k.y.a.)	PR^a^ (kg/cm^2^)	WC ^b^ (%)	Texture	Particle size (%)				Exp. implement no.
							Gravel^e^	Sand^f^	Silt^f^	Clay^f^	
I	Rice field	TLS	Modern	0.8	>71^c^	Silt loam	2.87	10.82	67.1	22.08	6002
II	Rice field	TLS	Modern	2.3	70.7	Silt loam	0.07	6.69	69.4	23.87	6013, 6002, 6003, 6015
III	Rice field	TLS	4.2–5.2	2.8^d^	62.7	Silty clay loam	0	0	72.1	27.9	6011
IV	Rice field	MS	4.5	4.7	33.7	Silty clay loam	0.1	2.6	69.3	28.13	6020
V	Habitation area	TLS	6.5	9.8	56.9	Silt loam	0.17	21.26	55.4	23.33	6021
VI	Xiashu Loess	MS	>6.8	22.6	22.9	Silty clay loam	0	5.17	61	33.85	6004
VII	Habitation and burial area	MS	6	26.4	21.1	Silt loam	0	17.91	65.4	16.7	6004
VIII	Paleo-coastal sediment	TLS	6–6.3			Silty clay loam	0	0.2	64.4	35.4	6012, 6014, 6009
IX	Deposit in a trench	MS	>5.3			Silt loam	0.07	11.98	65.4	22.66	6004, 6005, 6006, 6007, 6008
X	Habitation area	MS	4.2–5.2			Loam	1.21	34.9	44.5	20.6	1005
XI	Burial area	MS	4.2–5.2			Loam/clay loam	4.33	32.7	41.3	26.1	1004

*MS* Maoshan, *TLS* Tianluoshan

^a^PR is an indicator of soil hardness, quantified by how much force is needed to penetrate through a unit area of soil. PR values of fields VIII to XII were not measured due to unawareness of necessarily

^b^WC is the value of gravimetric water content, the ratio of the mass of water present in a sample to the mass of the sample after it has been dried to its constant weight. We collected soil samples for laboratory measurement of WC at the same time and in the same places where PR was measured. WC values of fields VIII to XII were not measured due to unawareness of necessarily

^c^The measured number was 68.1, but the actual WC was much higher because the soil was saturated with water flowing on the surface, and it was impossible to wrap the soil well with cling wraps with that much water, so the WC was reduced significantly when the soil sample was collected

^d^This PR value was originally measured at 1.75/16–4/16 kg/cm^2^ by a pocket penetrometer before a more reliable cone penetrometer arrived; the earlier figure was evidently inaccurate. A value of 2.8 was arbitrarily estimated afterwards according to the soil’s hardness compared to those in fields I, II, and IV, basically by multiplying the original results by 16

^e^Percentage of total soil

^f^Percentage of <2000 μm fraction

### Observations of Functionality

#### Edge Morphology and Soil Physical Property Matter

**Fig. 6:**
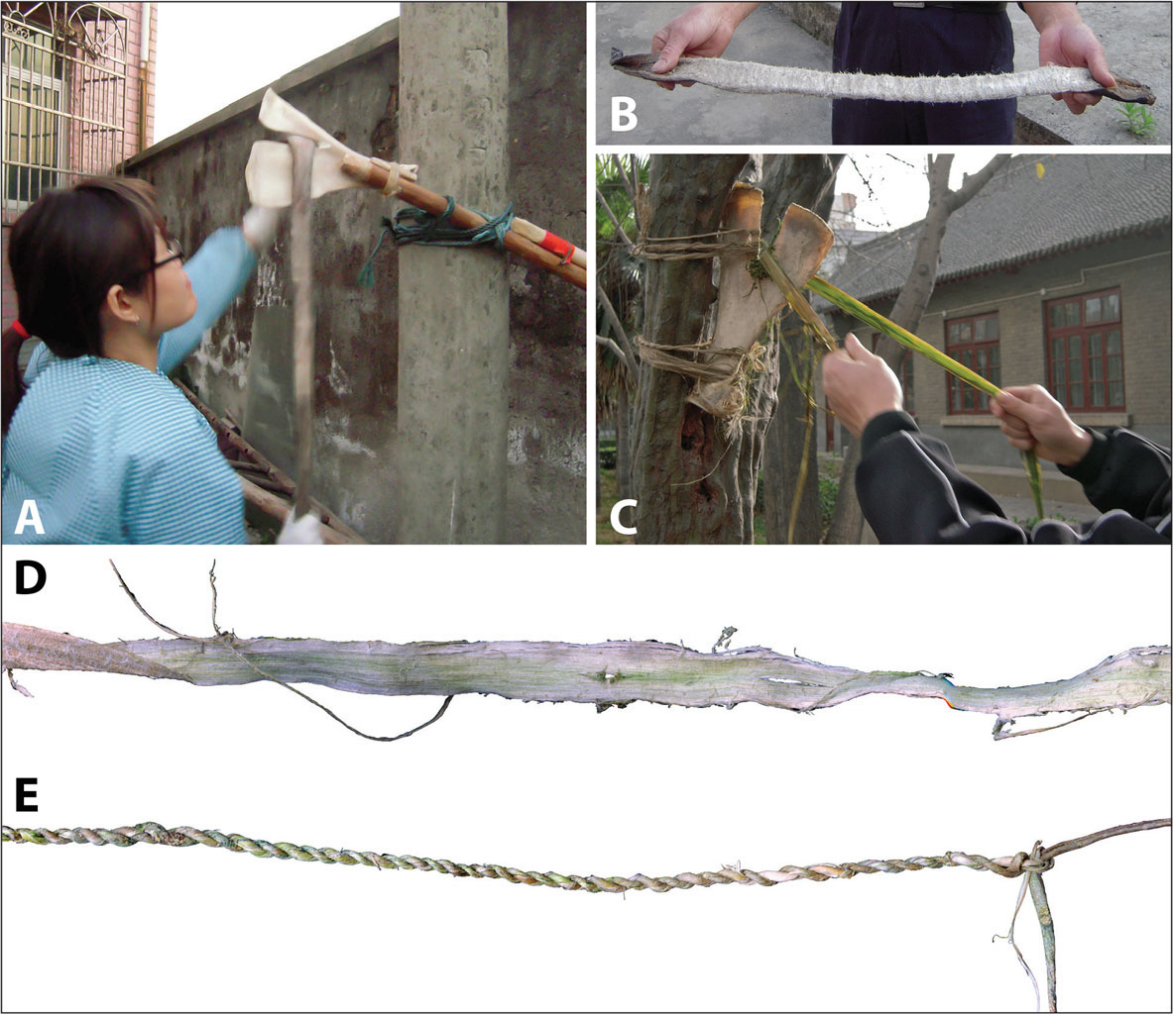
Experiments in using V-edged implements to soften hide strips and extracting fibers from bark. **a** Experimental implements no. 6017 processing a hide strip. **b** Hide strip after 1 h of processing. **c** Experimental implement no. 6018 processing a bark strip. **d** Bark strip after 10 min of processing. **e** Example of bark rope

Our experimental results suggest that V-edged tools very efficiently softened hide strips and assisted in the extraction of fibers from bark (Fig. 6) but were not suitable for penetrating earth or cutting plant fibers. Implement no. 6015 replicated a V-edged example at Tianluoshan, being hafted like a shovel, with sharpened, irregular edges along the inner margins of its two prongs as well as at the medial end of the bone. It was very awkward and barely functional for penetrating earth or clearing fields (cutting and removing weeds). A V-edged tool with straight inner margins, no. 6005, was able to penetrate earth in a soil substrate (field IX) similar to those in the Neolithic rice fields but was still awkward in comparison with the straight-edged implements that were used in the same field in the same way. All five participants who used no. 6005 complained that it was useful only when tilted toward its anterior side. By resting one foot on the glenoid cavity of the bone, the participants were able to push the implement harder so that the posterior prong of no. 6005 was able to penetrate the earth. However, when they tried to turn the soil over after breaking through it, as they necessarily would in tilling a rice field, most of the soil slipped through the space between the two prongs and remained unturned.

Hafted as shovels, straight-edged scapular implements with the Hemudu sophisticated hafting modifications penetrated soil well, especially in very soft substrates, and turned it over effectively. Scapular shovels barely functioned in very compact soils, such as undisturbed matrices and habitation and burial zones at Maoshan, penetrating only about 2 cm in depth with each stroke, and they wore down quickly (Xie et al. 2015). These results are consistent with previous digging experiments (*e.g.*, Ashbee and Cornwall 1961; Curwen 1926; Evans and Limbrey 1974) which showed that scapular shovels are functionally efficient only in sandy, light, moist, and/or uncemented soils and are inefficient in compact or well-cemented soils. Our experimental results specify this conclusion at a finer scale, suggesting that the Hemudu scapular implements can effectively penetrate earth only when the penetration resistance (PR) value of the soil is below 8 kg/cm^2^ and is best when it is below 4 kg/cm^2^ (Xie et al. 2015). Soil PR value is an indicator of soil hardness, quantified by how much force is needed to penetrate through a unit area of soil. In the Hemudu culture, only rice fields and habitation areas on the margins of the wetlands that are very moist would have had soils with PR values lower than 8 kg/cm^2^. Even in these soils, poor resistance to abrasion would still result in high consumption of scapular tools when using these implements to complete tillage in the 6.3 ha of the early Hemudu rice fields at the Tianluoshan site. For example, it would have required up to 200 scapular implements to accomplish the task in soil with a relatively low PR value of 4.5 kg/cm^2^ (Xie et al. 2015). Detailed discussion on the performance characteristics, especially time and energetic efficiency as well as material durability of scapular shovels in relation to soil physical properties, can be found in Xie et al. (2015).

**Fig. 7:**
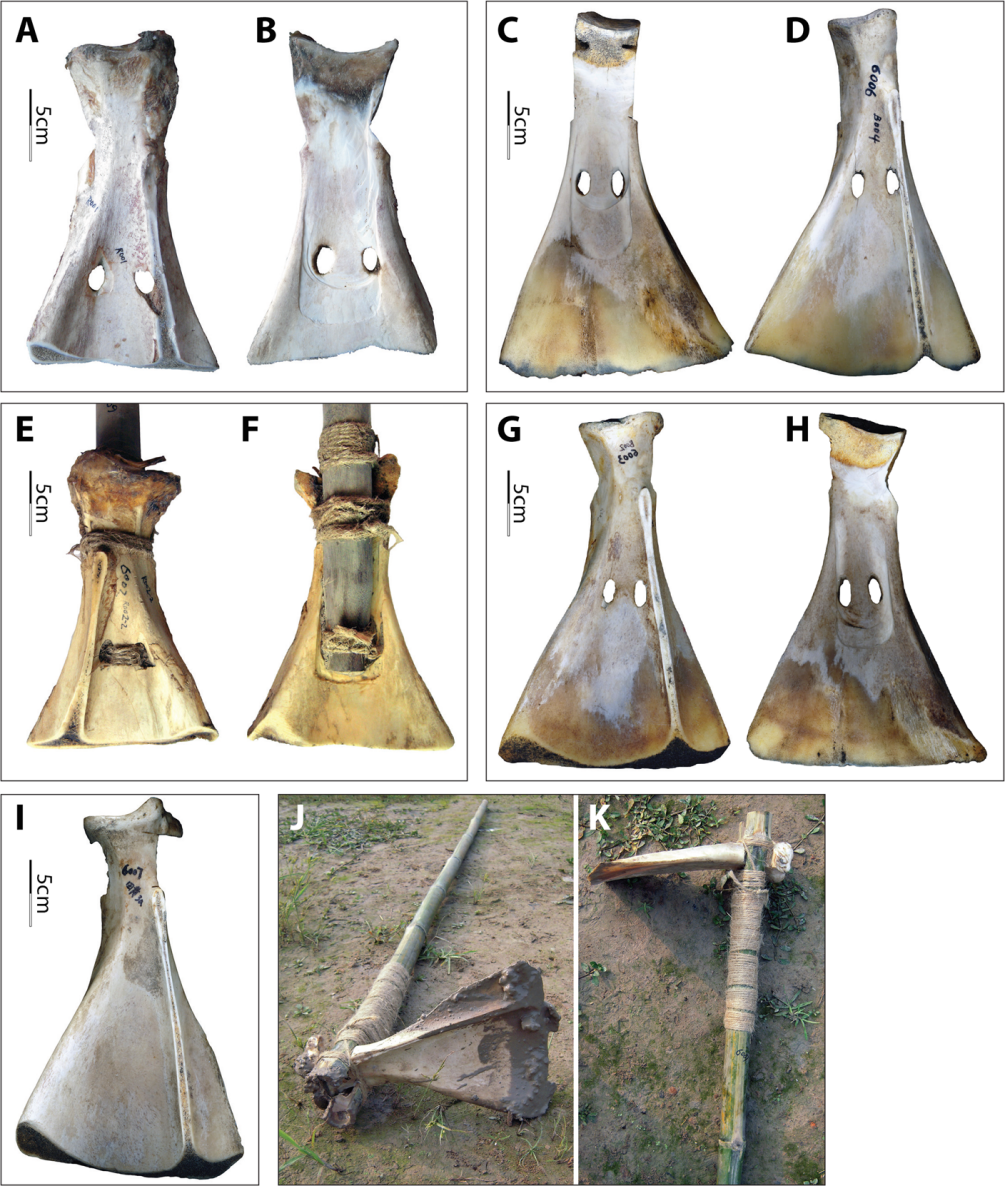
Examples of experimental implements. **a** and **b** Dorsal and ventral views of no. 6004. **c** and **d** Ventral and dorsal views of no. 6006. **e** and **f** Dorsal and ventral views of no. 6002. **g** and **h** Dorsal and ventral views of no. 6003. **i** Dorsal view of no. 6007. **j** and **k** Hoe hafting techniques used on no. 6007 and no. 6008

To efficiently penetrate even relatively soft soils, the spongy, weak, thick, uneven, and curved medial end of the scapula must be removed. Previous experiments have shown that hoes and shovels made from full-length bison scapulae were awkward and ineffective for digging (Davis 1965). Results of our experiments confirmed that a significant portion of the medial end needs to be removed to ensure a relatively straight blade for effective earth penetration. For example, we compared using no. 6006, the spongy medial end of which had been removed although the end was still slightly curved toward the edge, with no. 6004 (Fig. 7). For the same task (*i.e.*, breaking earth in the same field), no. 6006 required much more force than the shorter, straighter no. 6004.

We measured 59 modern cattle scapulae from animals aged 1 to over 20 years. The results show that with full lengths of 24.5–41.5 cm (mean = 34.7 cm, median = 36.2 cm), the maximum useful lengths of these scapulae, if they were to be crafted into earth-penetrating implements, ranged from 16 to 23 cm (mean = 19.8 cm, median = 19.7 cm).

#### Hafting Modifications and Modes Matter

The replicated implement with a vertical socket from the top for hafting (no. 6009) functioned as efficiently as implements with the Hemudu sophisticated groove-perforation-scored notch design when used as earth-penetrating implements in field VIII (paleo-coastal sediment). However, efficiency was measured only when the implements actually functioned. The shaft of implement no. 6009 occasionally separated from the implement during operation. Even though reinserting the shaft to the socket was quick and easy, it interrupted the work and would have been annoying to a worker.

Two implements were hafted as hoes (Fig. 7j, k). Crafted from a pair of scapulae sourced from one animal, no. 6008 utilized the maximum length of the straight bone, while no. 6007 was longer, retaining several centimeters of a curved portion at the medial end. Both were awkward to dig with and barely broke the earth, even though the soil in field IX, where they were used, was rice field-like, one of the softest soils in our experiments. This was due to the limited force that could be applied with such light-weight implements. However, tests showed that both implements were good for loading and moving loose soil. These experimental results are consistent with ethnographic observations by Wilson (1917) and Wang (1991), which suggested that scapular tools might have been too fragile to penetrate earth.

#### Additional Notes on the Functionality of the Hemudu Scapular Tools

Implement no. 6002 and no. 6003 were used to cut weeds and move loose materials around in field II. The results show that scapular implements could fulfill these functional needs. For cutting weeds, the shorter, straighter implement (no. 6002) was more effective, especially when the weeds were heavy or thick. For loading and removing loosened soils and/or weeds, the longer, more curved implement (no. 6003) was more efficient.

### Experimental Use-Wear Patterns

Experimental and archaeological samples were both washed gently under running water with mild soap and a soft brush and cleaned with alcohol-soaked cotton balls to remove fatty or greasy residues prior to microscopic examination. All experimental and archaeological specimens were initially examined with unaided eyes and the stereo-microscope (mostly with magnifications of ×8, ×10, and ×25) to gain a relatively complete overall picture of the use-wear patterns and to identify areas for subsequent examination at higher magnifications using an upright metallurgical microscope (mostly with magnifications of ×50, ×100, and ×200). With the stereo-microspore, we used an LED dual pipe illuminator; with the metallurgical microscope, we used a bright field, reflected light illuminator with a polarizing filter.^3^ We took use-wear photos with a Nikon Coolpix 4500 camera *via* the eyepiece tube, with the aid of a camera lens adapter for microscopes.

#### Use-Wear Patterns Derived from Processing Fiber

**Fig. 8:**
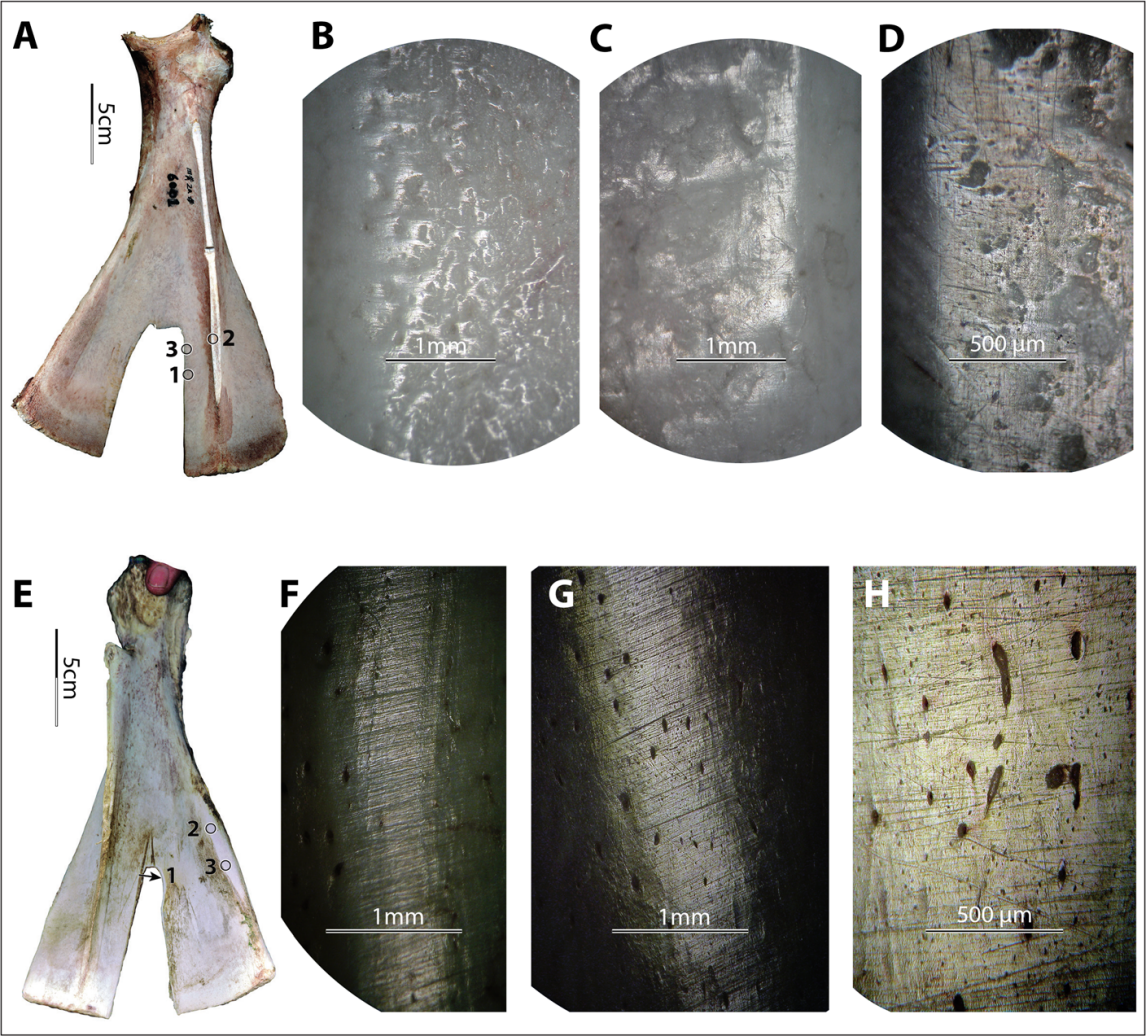
Examples of experimentally derived use-wear patterns of hide *vs.* bark. **a**–**d** Hide-derived wear, implement no. 6001, wear at spot 1 (×50), spot 2 (×50), and spot 3 (×100) after 60 min of use. **e**–**h** Bark-derived wear, implement no. 6019, wear at spot 1 (×50), spot 2 (×50), and spot 3 (×100) after 300 min of use. Note that (1) polish derived from processing hide is more rounding and invasive, (2) striations derived from processing bark are longer and better organized, and (3) striations both parallel and perpendicular to the direction of the prongs are present

Findings from hide and bark wear are consistent with results obtained from previous research. Flattening is apparent in bark-derived polish, in contrast to the invasive and rounding polish derived from processing more flexible hides. By invasive polish, we mean the wear that extends from the high points of asperities to their edges, which usually look rounded, and may even go down to the interstice. Striations derived from both bark and hide-processing vary in size, length, depth, and organization; however, bark-derived striations seem longer, somewhat finer, shallower, “better” organized, and of a more consistent size in general (Fig. 8). Image analysis software might be useful for more specifically quantifying the differences between the striations derived from bark and hide; in our analyses, their appearances do seem distinct from one and another. Hide-derived polish seemed brighter and smoother than bark-derived polish, based on our experiments; however, previous research has shown that plant-derived polish could be bright and smooth as well (*e.g.*, Stone 2011: Fig. 9.19). It is possible that these differences were a consequence of small sample size.

The directions of the striations should be consistent with the working direction of the tools. However, even though the principal action of the hide strip rubbing against the notch was mostly perpendicular to the direction of the prongs, the strip moved along the length of the prongs as well. The concomitant movements of left-and-right and up-and-down caused striations on both axes. Striations that parallel the elongated direction of the prongs are more common on the hide-processing experimental samples than on the bark-processing samples, as both V-edged scapular tools used for hide-processing showed many fine but clear striations parallel to elongated direction of the prongs (*e.g.*, Fig. 8d), while such striations were either absent or only very sparsely present on bark-processing tools (*e.g.*, Fig. 8h). However, it is not clear whether this was related to worked material or experimental duration or was just a coincidence of how the two sets of the experiments were conducted.

#### Use-Wear Patterns Derived from Penetrating Earth

Findings from soil wear appear to be more complicated. Because (1) soil substrate combines components with varying physical properties, such as sand, silt, clay, and organic matter, and (2) the percentage of each component varies from field to field, use-wear patterns derived from soils are diverse. To help with use-wear scrutiny, we developed expectations of soil-derived wear patterns based on our simplified understanding of tribology and previous use-wear observations.

Because sand and silt function as abrasive and shearing media when interacting with the penetrating implements, one can expect them to cause various sizes of striations and different degrees of flattening on the implements’ surfaces. Clay consists of much finer grains, so the main wear one expects to see on the implements is rounding and sheen (polish). Organic matter, especially plants, was expected to result in grouped striations and flattening. Because soil itself is highly plastic, one can expect friction to affect all surfaces of the portions of the implements in contact with the soil (*i.e.*, continuity) and can expect wear to be highly invasive.

Soil wear resulting from our experiments matched these expectations to some extent. It varied greatly from one field to another, but it all showed continuity and various sizes of striations. However, invasiveness was light or absent, which might have resulted from soil invading the interstices among asperities soon after contact with the tool surface and preventing the edges of asperities from experiencing further friction. Flattening was also light or absent, perhaps because soils in the experimental fields do not comprise much in the way of sand fractions (Table 5) compared to an effective abrading surface, and/or the sand was not cemented in hard materials to create an effective abrading substrate.

**Fig. 9:**
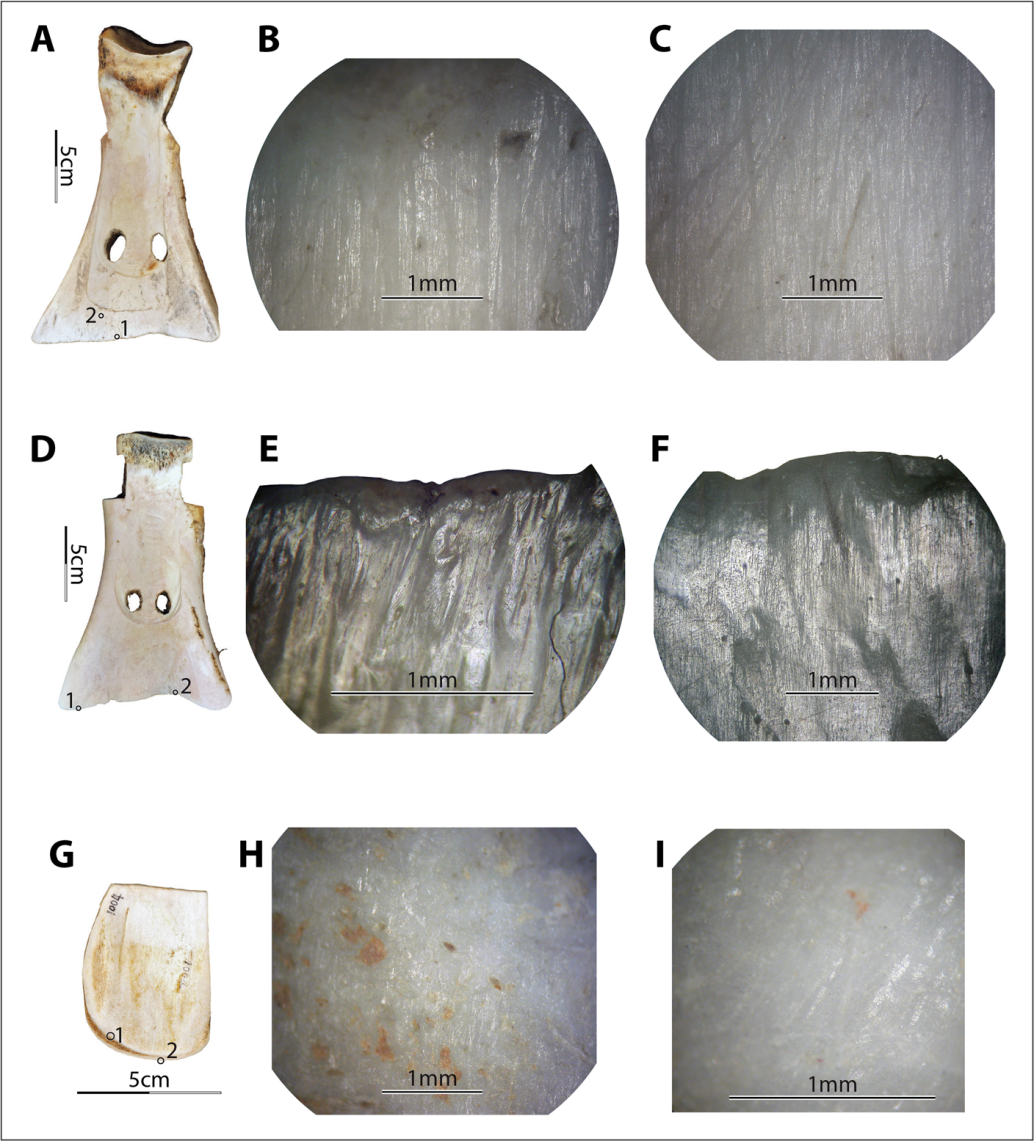
Variations in soil-derived wear patterns. **a**–**c** Implement no. 6004, wear at spot 1 (×50) and spot 2 (×50) of no. 6004 after 172 min of use in field no. IX. **d**–**f** Implement no. 6012, wear at spot 1 (×100) and spot 2 (×50) of no. 6012 after 102 min of use in field no. VIII. **g**–**i** Implement no. 1004, wear at spot 1 (×50) and spot 2 (×100) of no. 1004 after 90 min of use in field no. XI

Under the metallurgical microscope, we observed a mix of characteristics on each experimental specimen and highly diverse wear patterns across the specimens. As predicted, most specimens exhibited striations of varying widths and depths (*e.g.*, no. 6004; Fig. 9a–c), except those that were used either in very fine soil with little to no gravel or sand (*e.g.*, no. 6011, used in field III, and no. 6012 and no. 6014, used in field VIII; see Fig. 9d–f) or in very coarse soil with little gravel and a relatively high percentage of sand (*e.g.*, no. 1004, used in field XI; see Fig. 9g–i). Although perpendicular striations commonly appeared, slightly diagonal striations were dominant. The striation direction was consistent with the observed use modes of the tools. All participants in our experiments used the shovel to penetrate the earth at an angle, allowing the corner of the implement to lead the penetrating process, resulting in less resistance than would be produced by pushing the whole edge all at once straight down into the earth. Striations parallel or nearly parallel to the edge also appeared on a few samples, *e.g.*, no. 6004, used in field IX, caused by the users lifting the implements transversely to loosen the soil from the edge. Compared to the diagonal and perpendicular striations, the transverse striations are much finer and shallower because the pressure toward the bone blade was light (Fig. 9). The majority of the striations appeared well organized, with striations of similar sizes evenly distributed and roughly parallel to each other.

**Fig. 10:**
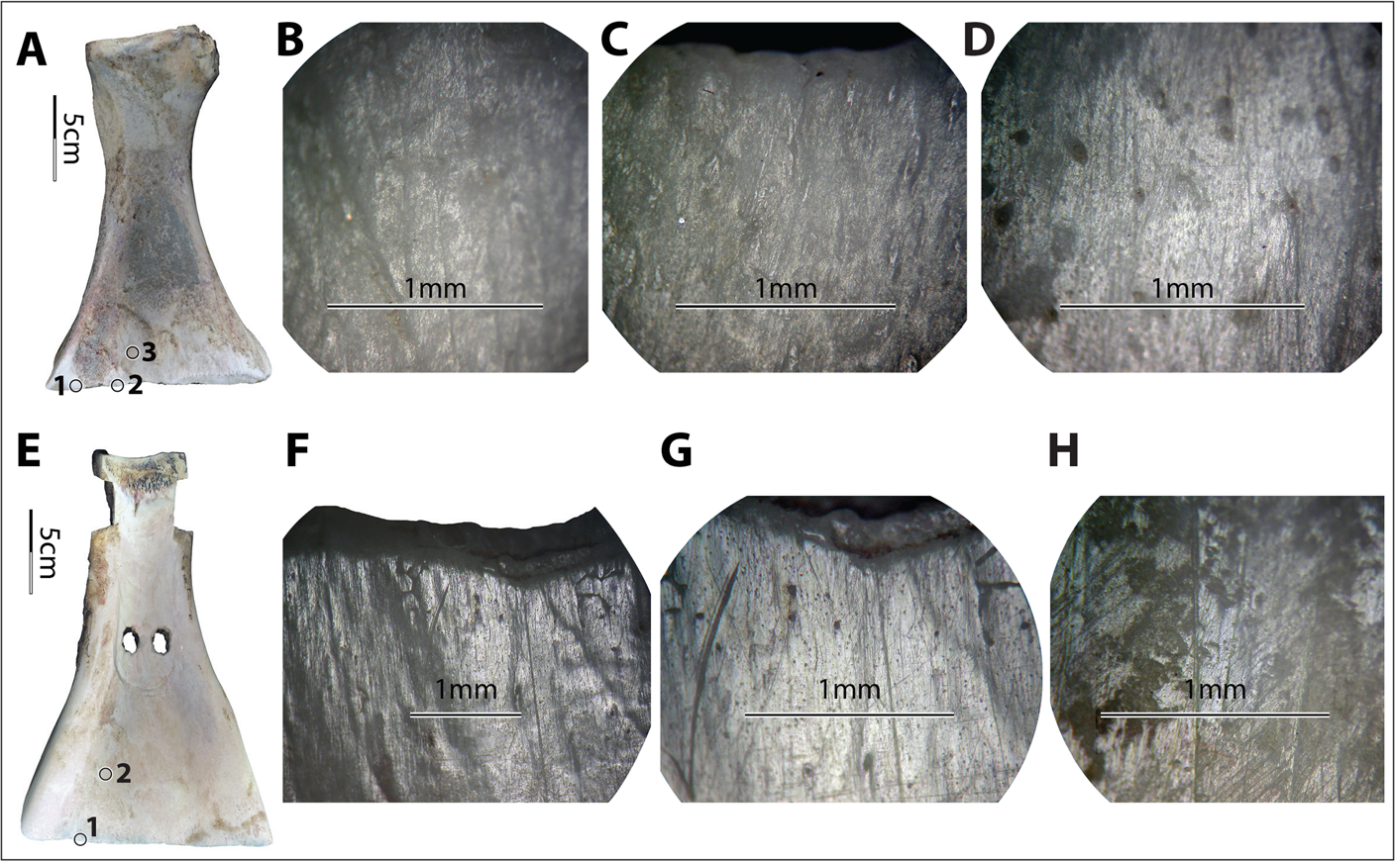
Diversity of soil-derived wear, perhaps due to variations in bone texture. **a**–**d** Implement no. 6009, wear at spot 1 (×100), spot 2 (×100), and spot 3 (×100) of no. 6009 after 83 min of use in field no. VIII. **e**–**h** Implement no. 6014, wear at spot 1 (×50, ×100) and spot 2 (100×) of no. 6014 after 84 min of use in field no. VIII

Polish derived from soils does not show consistent morphological patterns. For example, it is neither exclusively invasive nor exclusively flattening. Rather, it appears as both slightly invasive and rounded or slightly flattened (Fig. 9), depending on the soil condition. The texture and brightness level also vary greatly, from very rough to smooth and from matte to very bright. It seems that the finer the soil was, the smoother, brighter, rounder, and more invasive the polish it developed (Fig. 9). However, the conditions of the bones, including surface roughness and the presence of lubricants of fresh bone, also affected the polish pattern significantly, further complicating material-specific characterization. For example, no. 6009 was used under dry conditions, with a much coarser surface than no. 6012 and no. 6014, which were used fresh and very smooth. As a result, the polish on no. 6009 looked less smooth, less bright, less invasive, and much more flattened compared to the polish on no. 6012 and no. 6014, even though they were all used in the same soil for similar amounts of time (Fig. 10). Even at different spots on a single tool, use-wear could present in very different forms, *e.g.*, spot 1 *vs.* 3 on no. 6009 (Fig. 10).

**Fig. 11:**
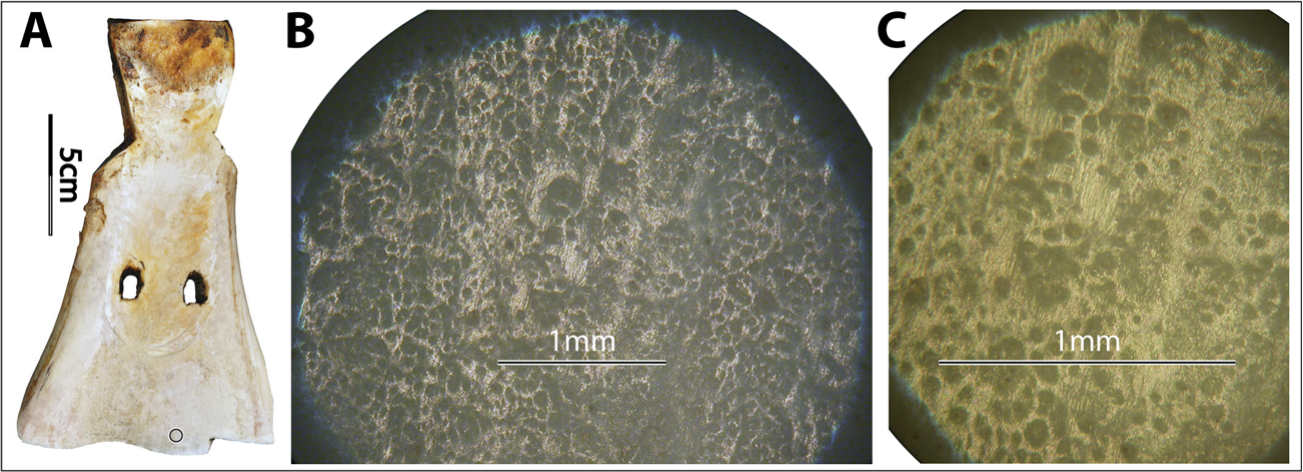
Example of how soil-derived wear can look like hide-derived wear. **a** Implement no. 6011. **b** and **c** Soil-derived wear (×50, ×100) at the indicated spot of no. 6011 after 8 min of use in field no. III

On bone, the morphology of soil-derived polish partially overlaps with that of hide-derived polish (*e.g.*, Fig. 11). Similar observations have been made in stone tool use-wear patterns: the micromorphology of soil-derived polish partially overlaps with that of dry hide- and wood-derived polish (Akoshima 1989: Table 1).

Since polish morphology is affected by so many variables and is extremely sensitive and diverse, it is not ideal for material-specific identification on bone tools. However, some soil-derived wear patterns still appear to be consistent, regardless of the great variety in detailed morphology. These patterns include (1) continuity, *i.e.*, the wear followed the contour of the implements’ surfaces and spread to both high spots and depressions almost evenly; (2) snap fractures; and (3) visible long striations of various sizes. In addition, the location and extent of the wear also provide significant clues for functional interpretation.

**Fig. 12:**
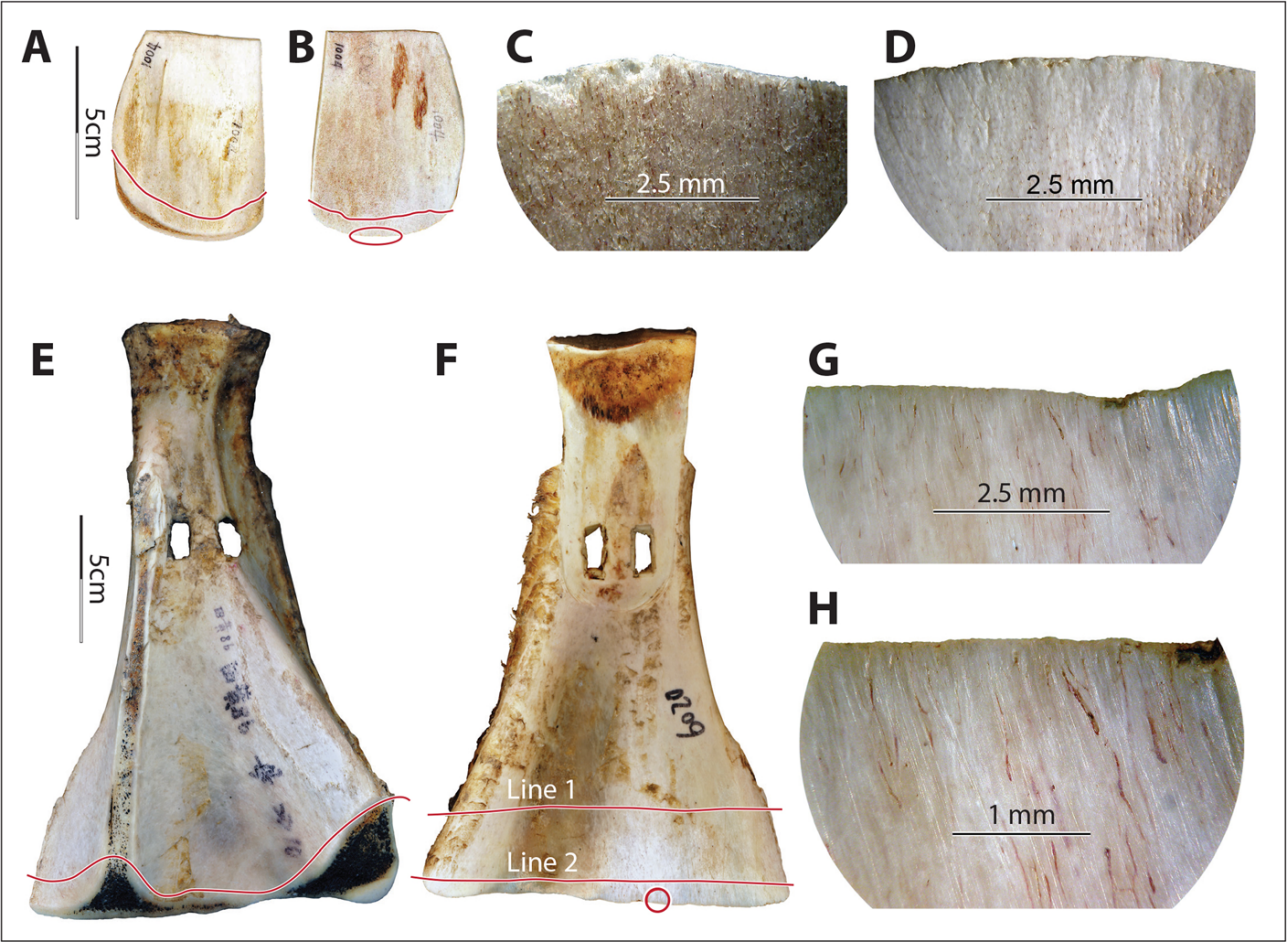
Soil signatures: continuity, snap fractures, visible long striations of various sizes, relatively large distribution of wear. **a** and **b** Dorsal and ventral views of implement no. 1004. **c** and **d** Implement no. 1004 before and after 90 min of use in field no. XI, photo (both ×10) taken at the circled area indicated in photo **b. e** and **f** Dorsal and ventral views of implement no. 6020. **g** and **h** Wear (×10 and ×20, respectively) at the circled area indicated in photo f after 26 min of use in field no. IV. Note that subpanels **d**, **g**, and **h** all show the presence of snap fractures, long striations of various sizes, rounding, and smoother surfaces resulting from use. The *lines* in subpanels **a**, **b**, **e**, and **f** show the distribution of soil-derived wear visible to unaided eyes. In particular, in subpanel **f**, *line 1* shows the distribution of wear and *line 2* shows the area where striations are most intensive. From the lines to the edges, use-wear fully covers the implements’ surfaces and has spread to depressions on the tools’ surfaces (*i.e.*, continuity)

The continuity of wear repeatedly appeared on the earth-penetrating implements across fields while it was absent on tools used for other tasks, granting distinctive characteristics to soil wear. The continuity of soil wear was made possible by the high plasticity of the matrix. Unlike the invasiveness of hide-polish, which extended from the asperities (high points) to their edges (which usually looked rounded) but barely extended to depressions on the tool surface, soil wear (including polish) usually did not reach the lower sections of the asperities but rather followed the contour of the implements’ surfaces and spread to depressions on the tools’ surface (Fig. 12).

Snap fractures appeared at the macroscopic level (Fig. 12). Twelve of 14 full-sized scapular earth-working tools with visible use-wear showed snap fractures on the edge, which were completely absent from hide- and bark-processing experimental tools. The absence of snap fractures on two experimental tools was probably a result of either use time or the presence of grass roots that caught on the edge of the implement and functioned as a cushion.

Long striations of various widths and depths also seemed unique to soil in our experiment. Macroscopically visible striations appeared on almost all earth-working tools (13/14 or 93 % of full-sized earth-working scapular tools showed macroscopic striations), but they also occurred on one out of four tools used for processing either hide or bark. However, the soil-derived striations are significantly longer, and their sizes vary greatly on a single implement. Therefore, simultaneous consideration of dimension and visibility of the striations on a macroscopic level helps with the identification of soil-derived wear (Fig. 12). Note that striations apparent to the unaided eye may be too coarse to be observed under a metallurgical microscope; consequently, the soil-wear signatures of visible long striations of various sizes are more reliably observed with low-power magnification or even with the unaided eye.

The location and extent of the wear also reflect the manner of work and provide important clues to differentiate soil wear from other kinds of wear. For example, compared to hide and bark wear, which appeared along the extreme margins of the tools, soil wear was present well away from the edge of the tool. However, these criteria are tool-specific and should not be applied without adjustment to identify soil *vs.* non-soil wear on bone in general. For example, striation length is significantly affected by the tools’ use modes, which are restricted by their morphologies, particularly the location of the working edge in relation to the rest of the implement. Just as with a needle penetrating through a hide, a deflesher scraping over the surface of a hide, or a hide strip rubbing against a V edge, the extensions of wear are influenced by both the direction of the dominant motion and the overall form of the implement. One must also be very cautious when applying these criteria to identify functions of the same tools in the archaeological collections, because wear on archaeological samples is much more complicated. The actual use of those tools will probably not have been as exclusive as the replicas used under experimental conditions, or the wear might have been altered by a variety of processes including weathering, post-deposition, and post-excavation treatments.

## Identifying Scapular *Si* Tools in Hemudu Culture

None of the archaeological scapular tools from Hemudu or Tianluoshan show use-wear patterns identical in morphology to any of those derived from soil in our experiments. However, when we jointly apply the criteria of (1) continuity, (2) snap fractures, and (3) visible long striations of various sizes, as well as (4) distribution of wear and (5) edge location in relation to the rest of the tools, many archaeological tools show soil-like wear (Table 6). Depending upon the situation, an implement not always bear snap fractures to be confidently identified as an earth-working tool, because such traces were distributed exclusively along the tool’s extreme edge and could be obscured relatively easily by secondary uses and edge resharpening.

**Table 6:** Hemudu and Tianluoshan archaeological specimens with soil-like wear

Site	Collection no.	Species	Weight (g)	Length (cm)	SLC (cm)	Edge style	Edge angle (°)	Use-wear resembles
HMD	272	W.B.	213.5	19.5	6.13	E	S16, rest rounded	6013
HMD	278	W.B.	354^a^	22.9^a^	6.93^a^	Undet	–	6004, 6009
HMD	279	W.B.	192^a^	19	7.25^a^	EV	13–15	6012, 6014
HMD	280	W.B.	196^a^	19.4	6	E	S23, rest too thin to measure	6012, 6020
HMD	271	W.B.	235	17.2	6.05	E	29–32, S32	6002, 6004
HMD	275	W.B.	202	13.5	6.82	E	53–60	1004, 6004
HMD	276	W.B.	190	18.4	6.12^a^	E	A16, S25, rest rounded	6002
HMD	284	W.B.	166^a^	17^a^	5	Undet	–	6020
HMD	281	W.B.	228	16.4	6.35	E	33–36, A47	6013
HMD	282	W.B.	186^a^	16.4^a^	–	E	S14, A20, rest too thin to measure	6009, 6012
HMD	283	W.B.	218^a^	18.7	–	EV	A17, rest rounded	6020
HMD	290	W.B.	208	16	6.58^a^	EV	36–41	6002, 6004
HMD	295	W.B.	235^a^	20.2	–	E	35, A17	6004
HMD	298	W.B.	204^a^	17.3	–	EV	38, S24	6009
HMD	302	W.B.	233^a^	20.6	–	EV	30, S14	6005, 6020
HMD	304	W.B.	266^a^	23	7.35	EV	S14, rest rounded	6009, 6012
HMD	306	W.B.	168^a^	18.6^a^	6.37	EV	S18, A14, rest rounded and thin	6002, 6011, 6009
HMD	307	W.B.	137^a^	18^a^	4.65	S	–	6002
HMD	314	W.B.	297^a^	23.7^a^	6.9^a^	EV	S19	6002
HMD	323	?	90^a^	13.5^a^	5.25^a^	EV	28, S21–23	?
HMD	325	Deer	116^a^	18.2^a^	4.37	Undet	–	6002, 6013
HMD	326	Bear	393^a^	25	–	E	40–45	6004, 6005
HMD	328	W.B.	286	18	6.55^a^	E	32–38	6011?
HMD	330	W.B.	335	20.4	6.25	EV	20–25	6020, 6012
HMD	338	W.B.	304	22.7	7.46	EV	S34, rest rounded	6002
HMD	339	W.B.	202	17.8	6.75	E	30, S21-28, A25	6011
HMD	340	W.B.	194	15.4	7^a^	E	S23, A30	?
HMD	342	W.B.	337^a^	30.5	6.22^a^	ES	S15	6021
HMD	344	W.B.	195^a^	16.8	6.67	Undet	Rounded	6011, 6005
HMD	350	W.B.	220	17.5	6.12	E	28	6002, 6004
HMD	352	W.B.	177^a^	14.1^a^	–	EV	40, S44, A44	6012, 6014
HMD	353	?	112^a^	15.8^a^	–	Undet	Rounded	6005
HMD	354	W.B.	158	14.9	6.58	E	39, A42, S32	1005
HMD	360	W.B.	243^a^	17.5	7.13	Undet	A64, rest missing	6002, 6012
HMD	362	W.B.	144^a^	11.2^a^	–	E	43, A26, S26	6012
HMD	363	W.B.	198	16.5	7.12^a^	EV	20–24	6012
HMD	365	W.B.	298	18.7	6.86	EV	S15	6012
HMD	288	Deer	113^a^	15^a^	3.6	Undet	–	6009, 6021
HMD	336	Deer	110	20.4	3.34	V	Rounded	6020
TLS	T103(5):80	?	144^a^	23.2	–	Undet	Rounded	6020
TLS	T206(5):1	W.B.	191	15.8	5.94	E	29–41	6004, 6021
TLS	DK3(7)	W.B.	247^a^	17	6.61	Undet	39	6020, 6021
TLS	DK3(7):8	?	56^a^	14.4	–	Undet	15	6011, 6021
TLS	T203(3)A:1	W.B.	232	15	6.55	E	34–39	6020, 6021
TLS	T301(5):4	?	66^a^	9.6^a^	–	Undet	–	6012
TLS	T404(5)	W.B.	252^a^	17.3	6.21^a^	EV	S26, rest rounded	6002, 6004
TLS	T307(5)	W.B.	168^a^	13.7	5.72	Undet	–	6002, 6004
TLS	T203(7)	W.B.	289	21.2	6	EV	25	6021, 6011
TLS	T104(3):SGS1 and T005(3):SGS1	?	84^a^	12^a^	–	Undet	22–25	6021, 6011
TLS	IIT403(5):5	W.B.	235^a^	19	–	Undet	18–20	6021

Alike the edges of the replicated samples, edge angles of the archaeological samples vary. In the column of “edge angle,” pure numbers or number ranges indicate the angles of most of the edges, the numbers following an A are the angles on the anterior borders, and the numbers following an S are the angles on the spines. Edge style indicates both edge locations and morphologies

*W.B.* water buffalo, *SLC* width of the scapular neck, *E* straight edge at the medial end of the bone, *S* at the side of the bone, edge may or may not be straight, *V* two-pronged, *ES* coexistence of E and S edges, *EV* coexistence of E and V edges, – the portion was missing, *Undet* undetermined

^a^Measured unit was incomplete or broken

We identified soil-derived and/or fiber-derived wear patterns on 80 out of 155 archaeological scapular implements. Among the 80 scapular implements with identified wear, 50 are interpreted as having been used for earth-working, including 39 out of 93 from Hemudu and 11 out of 62 from Tianluoshan. The main differences between soil-like wear on the archaeological artifacts and soil-derived wear on the experimental pieces are the brightness and texture of the observed polish. Polish on the archaeological samples bearing soil-like wear appeared smoother and brighter in general than the soil polish generated in our experiments. As shown in Figs. 15 and 16, some of the archaeological samples also bear polish visible to unaided eyes all over the surface; we neither saw nor imagined seeing this result of use on our experimental samples.

Hypothesized causes for such differences include but are not limited to (1) each of the archaeological earth-working implements having been used in a variety of soils, and even non-soil contexts, so that the wear was derived from a mix of contexts, unlike the relatively “pure” working context of the experimental samples; (2) the possibility that archaeological samples (rather than other earth-penetrating tools) might have been used as less-damaging soil-collecting tools in loosened substrates; (3) the archaeological samples having been deliberately polished; and (4) possible wiping, cleaning, or spit polishing the tools,^4^ multiple usage, curation, transport, and storage by the users, as well as post-depositional processes that may have altered the original use-wear. The results of our research suggest that at least hypotheses 1 and 2 are not only possible but probable. Future experiments and analyses are needed to evaluate the remaining hypotheses.

Table 6 presents the 50 archaeological samples which exhibit soil-like wear—their source animals, dimensions, and edge morphologies—and the experimental specimens whose wear most closely resembles the wear on each archaeological sample. The use-wear similarities were based mainly on the sizes and densities of striations, allowing us to discuss the use contexts of the earth-working implements in the Hemudu culture.

Forty of the 50 Hemudu culture earth-working implements are crafted from scapulae of water buffalo, three from large deer, and one from a bear: the source animals of the remaining six implements were unidentified because the diagnostic portions were missing.

Water buffalo scapulae have longer potential use-lives than deer scapulae because of their concomitant larger dimensions and greater robustness, which are ensured by their thicker cortical bone. The three deer scapulae that were used for earth-working are relatively large and robust compared to other deer scapulae but still smaller than the smallest water buffalo scapula that was crafted into an earth-working implement (Table 6). The only modified bear scapula is larger and more robust than any of those of water buffalo (Table 6). However, the blade curves and the overall shape are less convenient to modify into a shovel than a water buffalo scapula, and the implement looks awkward for earth-working. The four exceptional scapular implements made from bear and deer may represent children’s shovels and/or reflect a short supply of the most suitable raw materials. Additional evidence from the unmodified faunal record supports the short-supply hypothesis (Xie 2014, pp. 200–207). Future research can test the children’s shovel hypothesis.

The basic dimensions, counting only intact specimens for the weight and intact portions for the length and SLC (*i.e.* the width of the scapular neck), of the Hemudu earth-working implements made from water buffalo scapulae are listed in Table 7. The identified earth-working implements from the archaeological collections are 13–30.5-cm long. The shortest archaeological sample (13 cm) was crafted from one of the largest and most robust water buffalo scapulae. According to experimental observations, 13 cm probably represents the minimum functional length of earth-working implements. Considering the dimensions and morphologies of modern cattle scapulae, the original length of this implement is estimated to have been 23 cm if it was made for breaking ground, suggesting that approximately 10 cm was worn off the length from use. The longest archaeological sample (30.5 cm) includes an extended curved portion toward the edge, not ideal for earth penetration; it was likely used for moving soil rather than breaking ground (see below).

**Table 7:** Earth-working scapular tools crafted from water buffalo scapulae

	Minimum	Maximum	Mean	Median	*N*	SD
Weight (g)	158	335	232	220	19	48
Length (cm)	13	30.5	18.2	17.5	35	3.4
SLC (cm)	4.7	7.5	6.4	6.6	34	0.6

Most archaeological use-wear resembles the wear generated by relatively soft, fine soils in rice fields, or soils of similar textures (*e.g.*, fields I, II, IV, VIII, and IX), and the implements showing this kind of use-wear may represent the *si* agricultural tools. Far fewer of the archaeological samples showed the kind of use-wear derived from harder, coarser soils in the contexts of habitations and graves (*e.g.*, fields V, X, and XI). To get a rough sense of the working contexts, particularly agricultural and non-agricultural uses of the implements, we developed a method to evaluate the relative frequency of earth-working by scapular tools in these contexts. We assigned scores to the identified working context(s) of each implement and summed up the total scores in each context for comparison. For example, if the soil-like wear on an archaeological implement resembled the wear on one or more experimental implements that were used in rice fields (either ancient or modern, *e.g.*, wear on HMD 271 resembled that on no. 6002 and no. 6004 used in the modern rice field of the Tianluoshan village and the ancient rice field at Maoshan, respectively), one point was assigned to the rice field. If the soil-like wear of the archaeological implements resembled wear on more than one experimental implements used in both agricultural and non-agricultural contexts (*e.g.*, wear on TLST203(7) resembled that on no. 6011 and no. 6021, used in an ancient rice field and a habitation area at Tianluoshan, respectively), 0.5 point was assigned to each context. The total scores for each working context at Hemudu and Tianluoshan are listed in Table 8.

**Table 8:** Working contexts of the earth-working implements

	Agriculture	Coastal sediment	Others	Sample size
HMD	24.5	10.5	3	38
TLS	6	1.5	3.5	11

The results suggest that scapular earth-working implements were used more frequently in agricultural fields than in non-agricultural areas. At Hemudu, the frequency of using earth-working scapular tools in agricultural contexts is estimated to be about eight times that in non-agricultural contexts (24.5 *vs.* 3 points). At Tianluoshan, the frequency of non-agricultural use of implements was much higher; however, it is still lower than in agricultural fields (3.5 *vs.* 6 points). The paleo-coastal sediment shares similar soil properties with the agricultural fields and the sediments on which the earliest pile-dwelling houses at Tianluoshan (and probably Hemudu) were constructed (Xie et al. 2015); therefore, earth-working implements with coastal-sediment-derived wear could have been employed in either agricultural or non-agricultural contexts. Even when counting paleo-coastal sediment exclusively as a non-agricultural context, the frequency with which implements were used in agricultural fields at Hemudu was still higher, although at Tianluoshan, with a sample size of 11, the difference between six implements in agricultural fields and five in non-agricultural fields is statistically negligible.

Such rough estimates of use frequencies may not represent the exact circumstances of the tools’ use; however, the overall picture derived from these numbers should be reliable. The estimated working contexts at Tianluoshan may be closer to the actual situation, because half of the experiments were conducted at fields at this site while none were carried out around the Hemudu site.

Analyses of the performance characteristics of scapular implements in different soils show that they penetrated earth relatively well and were relatively durable in agricultural and similar soil substrates. In contrast, in non-agricultural fields where soils are usually harder and coarser, such as field V in the habitation area of Tianluoshan, they were much less effective and wore down rapidly. Detailed discussion on performance characteristics can be found in Xie et al. (2015).

**Fig. 13:**
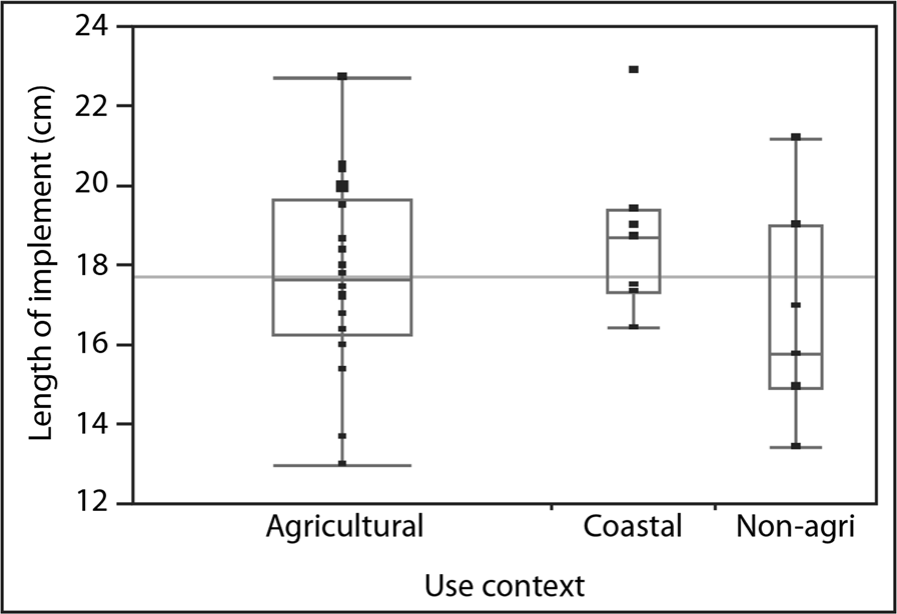
Comparison of median length of implements employed in agricultural soil, coastal sediment, and non-agricultural soil (sample sizes 18, 7, and 7)

Although none of our experimental implements were used to the extent that breakage occurred, presumably those used to *penetrate* non-agricultural soils would be more subject to breakage because they had to absorb more force. To reduce the risk of breakage and also enhance the implement’s penetration ability, shorter implements with narrower edges (as the width of the bone decreases toward the glenoid cavity) in non-agricultural, compact soils may be most effective. Also, because scapular implements became exhausted much more quickly from *penetrating* non-agricultural, more compact soils, one might reasonably expect them to be shorter and exhibit higher percentages of broken implements when compared with those used to penetrate agricultural soils. However, the archaeological samples did not match these expectations (Fig. 13 and Table 9).

**Table 9:** Lengths (cm) of archaeological earth-working implements crafted from water buffalo scapulae at Tianluoshan and Hemudu

Context	Complete length (cm)						Number of broken samples
	Max.	Min.	Mean	Median	*N*	SD	
Agricultural	22.7	13	17.8	17.6	18	2.4	3
Non-agricultural	30.5	13.5	18.4	16.4	8	5.5	0
Non-agricultural (outlier removed)	21.2	13.5	16.6	15.8	7	2.7	0
Coastal sediment	23	16.5	18.8	18.7	7	2.1	5

The longest earth-working implement crafted from water buffalo scapula in the archaeological collections (30.5 cm), with a long curved portion toward the edge, exhibited soil wear from a non-agricultural context. Even after this outlier is removed, the average and median lengths of the 18 implements that were used only in agricultural soils and remain unbroken lengthwise are only 1.2–1.8 cm longer than the seven implements used in non-agricultural contexts. The two sample *t* test results, *t* (1.2) = 1.12 and *p* = 0.3567, suggest that this difference is not significant. The percentage of broken implements among the agricultural implements is 14.2 % (or 3 out of 21), while no broken implements crafted from water buffalo scapulae were identified as having been employed in non-agricultural context. Collectively, these clues may indicate that scapular implements used in non-agricultural contexts were more likely to have been used for moving loosened soils; if they were for breaking ground, they must have been used only when the ground was very moist and therefore much softer. This argument does not assume that a tool was used exclusively in either agricultural or non-agricultural contexts. In fact, most implements with soil wear similar to that derived in non-agricultural soils also possess agricultural soil-like wear. It is possible that the samples that exhibited only wear from agricultural soils had been used in non-agricultural soils but that initial wear was obscured by later use. Even if it is not completely obscured, non-agricultural soil wear, especially that generated by penetrating compact soils, is more difficult to capture by use-wear analysis because its distribution is limited to areas close to the edges due to shallower penetration. These possibilities do not conflict with the overall picture drawn by experimental and ethnographic research, however. Scapular implements may have been used more commonly in agricultural soils as *si*. Although also used in non-agricultural contexts, that use was directed toward lighter tasks given the limitations of scapular implements’ functional feasibility and material durability.

Overall, it seems safe to conclude that most, if not all, scapular implements exhibiting soil-derived wear represent the *si* implements, and these implements were used in both agricultural and non-agricultural contexts.

## Non-*Si* Scapular Tools in the Hemudu Culture

Thirty archaeological samples exhibit fiber-like wear from processing plant and/or animal tissues (Table 10). The identifications of non-soil worked material were based on the micro-morphologies of polish and striation as we discussed in previous section of this article. For example, longer, finer, and better-organized striations and flattening polish are indicators of plant fiber processor, while invasive and rounding polish is usually from processing animal tissues.

**Table 10:** Hemudu and Tianluoshan archaeological specimens with fiber-like wear

Site	Collection no.	Species	Weight (g)	Length (cm)	SLC (cm)	Edge style	Worked material
HMD	274	Deer	131.2	21	3.51^a^	ES	Hide
HMD	277	W.B.	215.2^a^	20.5	6.05^a^	EV	Hide
HMD	285	Deer	112.5	19.6	3.39	EV	Hide?
HMD	286	Deer	173.8^a^	27.14	3.75	Undet	Hide
HMD	289	W.B.	226.5^a^	18.1	–	EV	Plant
HMD	299	W.B.	216.7	18.8	6.02	EV	Plant?
HMD	301	W.B.	225.2^a^	21.95	–	ES	Plant?
HMD	303	W.B.	245.3^a^	25.74	6.65	EV	Hide
HMD	305	Deer	109.9^a^	21.22^a^	3.97^a^	V	Hide
HMD	309	Deer	175.9^a^	22.84	4.16	V	Plant?
HMD	312	Deer	122.3^a^	22.5	3.3	Undet	Plant?
HMD	313	Deer	159.8^a^	23.91	4.6	V	Plant
HMD	315	Deer	163.3^a^	19.74	3.9	V	Plant or hide
HMD	316	Deer	198^a^	23.7	Undet	EV	Hide
HMD	317	Deer	115.2^a^	25.71	–	Undet	Plant
HMD	322	?	82.8^a^	14.43^a^	4.59	EV	Hide + plant?
HMD	327	W.B.	280.5^a^	26.78	6.2^a^	EV	Plant
HMD	332	Deer	160^a^	25.35	3.76	V	Plant?
HMD	333	W.B.	407.6	26.62	6.92	EV	Plant
HMD	337	Deer	140.5	20.3	3.7^a^	EV	Plant or hide
HMD	355	W.B.	233.1^a^	18.6	6.5	E	Plant?
HMD	356	Deer	106.3^a^	19.87	3.45^a^	ES	Hide
HMD	359	W.B.	302.2^a^	22.38^a^	6.81	Undet	Hide?
HMD	1577	?	32.7^a^	6.94	n/a	Undet	Hide + unidentified
TLS	K3(6):28	?	25.5^a^	8.9	–	Undet	Plant
TLS	T003(6):3	?	47.5^a^	12.47	–	EV	Plant?
TLS	T105(5):3	Deer	191.8^a^	21.2	3.96^a^	V	Plant
TLS	T105(5):5	?	23.2^a^	6.42	–	Undet	Plant?
TLS	T301(7):1	?	29.8^a^	6.6	–	Undet	Plant
TLS	T302(4):4	W.B.	228.6^a^	16.2	7.52	Undet	Plant?

Edge style indicates both edge locations and morphologies

*W.B.* water buffalo, – the portion was missing, *SLC* width of the scapular neck, *E* straight edge at the medial end of the bone, *S* at the side of the bone, edge may or may not be straight, *V* two-pronged, *ES* coexistence of E and S edges, *EV* coexistence of E and V edges, *Undet* undetermined

^a^Measured unit was incomplete or broken

Although the number (30) of scapular implements identified as fiber processors is smaller than the number (50) of identified as *si*, this may reflect our lack of experience and confidence in our identification of the first group rather than the real relative abundance of the two tools. Even so, the data collected from all of these scapular implements with identified functions allowed us to understand the morphological designs and raw material choices of the *si* agricultural tools in a comparative manner.

## The Morphologies of the Hemudu Scapular *Si* Implements

### Modifications for Hafting

**Fig 14:**
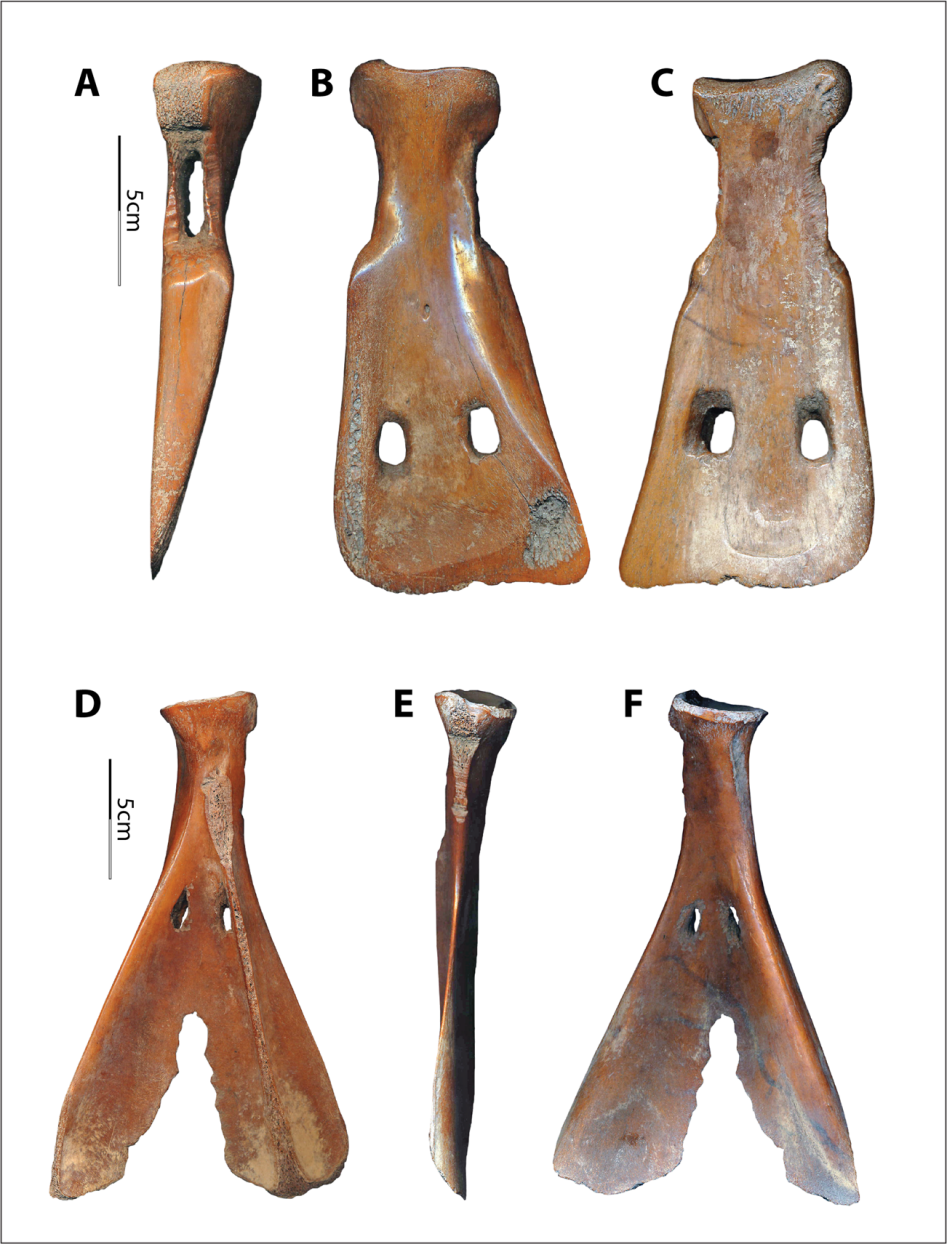
Hafting designs of Hemudu scapular earth-working implements. **a**–**c** Typical Hemudu hafting design for earth-working implements crafted from water buffalo scapulae: lateral, dorsal, and ventral views of HMD 271. **d**–**f** Hemudu hafting design for implements crafted from deer scapulae: dorsal, lateral, and ventral views of HMD 336. Note that HMD 336 lacks (1) a deep hafting groove on the ventral surface, (2) deep notches on the sides of the neck, and (3) modifications around the glenoid cavity. Also, the neck of HMD 336 is not scored through as seen in HMD 271 (compare **a** with **e**)

The Hemudu *si* exhibits sophisticated modifications for hafting (Fig. 14). All but two implements have a hafting groove, two perforations, and significant removal of the cortical bone on the lateral sides of the scapular necks. In addition, both sides of all but five implements are transversely scored through. Two of the five exceptions are crafted from water buffalo scapulae and the remaining three from deer scapulae. In both of the exceptions that are crafted from water buffalo scapulae, the cortical bone on the lateral sides of the neck is removed to a degree comparable with the samples that are scored through. In contrast, the degree of modification of the necks of the three deer scapular implements is much lower, with only one side slightly notched (Fig. 14). Moreover, projecting portions around the glenoid cavities were flattened to ensure effective lashing. Again, the modifications around the glenoid cavities are much more extensive on the scapulae of water buffalo than on those of deer.

As previously mentioned, these hafting modifications and the presence of lashing materials on two scapular implements suggested they had been used as shovels (see Fig. 4). Results of our use-wear analyses locate hafting wear on the neck surfaces of the implements, confirming that the tight fastening method illustrated in Fig. 3 was commonly employed. Such hafting designs are much more elaborate than those of the scapular tools used for other purposes in the Hemudu culture. They are also more elaborate than hafting designs on scapular earth-working implements from other world regions.

**Fig. 15:**
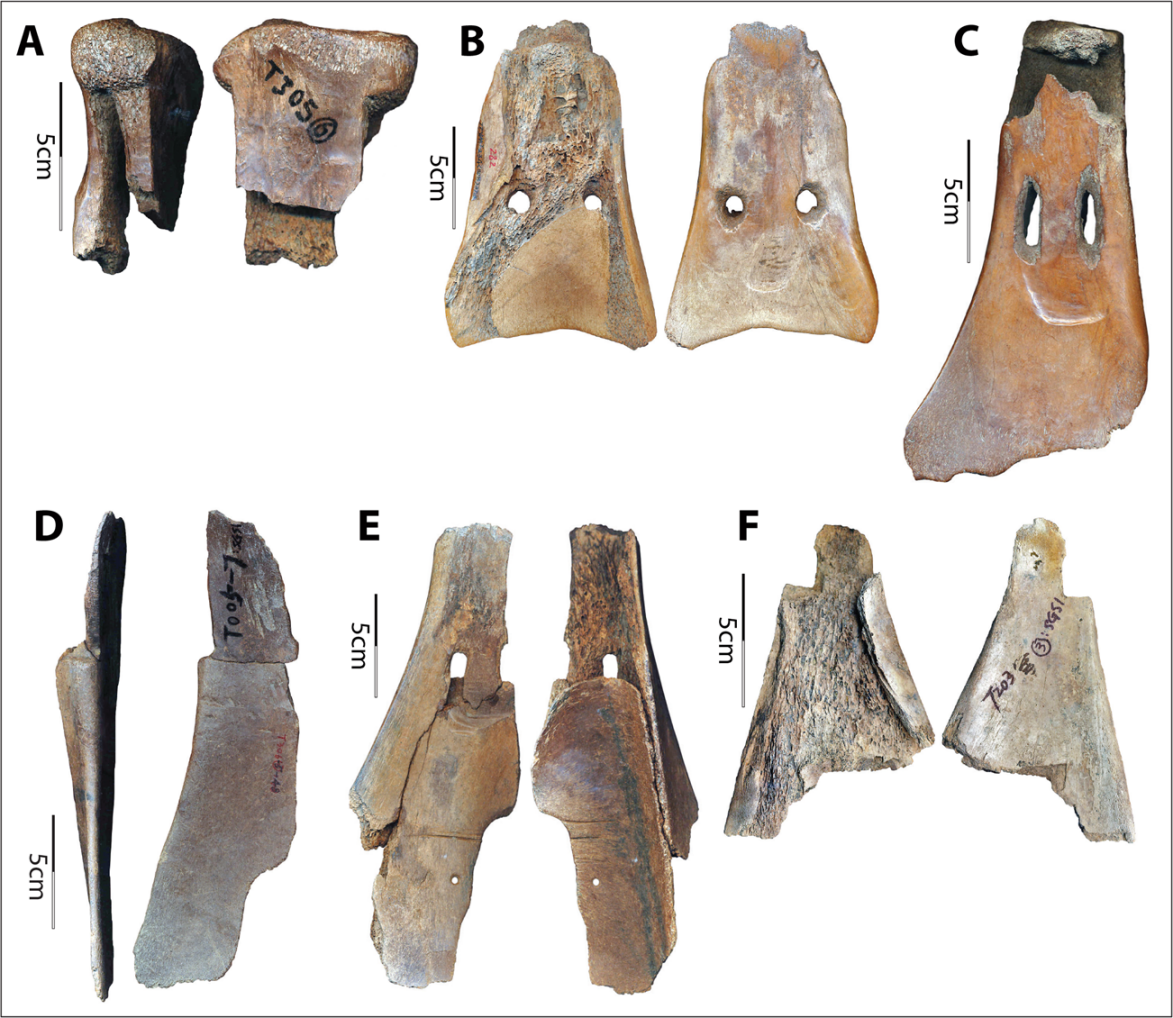
Breakage patterns of Hemudu scapular earth-working implements resulting from hafting modifications. **a** TLST305(6):SGS1 (lateral and ventral views). **b** HMD 282 (dorsal and ventral views). **c** HMD 280 (ventral view). **d** TLST306(5):48 and T005(7):SGS1 refitted (lateral and ventral views). **e** TLST103(5):80 (ventral and dorsal views). **f** TLST203(3):SGS1 (dorsal and ventral views)

Compared with other hafting designs, these Hemudu hafting modifications may fasten the blades to the handles much more securely. However, from a pure hafting perspective, these Hemudu hafting modifications exceed usual standards. The complete removal of the strongest portion (all cortical surfaces) on the sides requires much more effort in manufacturing and significantly weakens the scapular necks. Transversely scoring through the necks exacerbates this problem, causing breakage patterns common in the archaeological collection (Fig. 15). These overdesigns (*i.e.*, designing in a manner that is excessively complex that exceeds usual standards or minimal needs), which emphasize tight fastenings, indicate that the earth-working tasks at Hemudu culture sites may have been very arduous, probably because the soils were more difficult to penetrate or stickier (thus, blades would have stuck in the soil easily) than those in other areas of the world where scapular tools were employed and/or because the task was extremely intensive and did not allow much interruption for rehafting.

Notches on the sides of a few implements from Hemudu are shallower and unscored, or deep but unscored, or scored just half-way through. These may indicate experimentation with a strategic design process resulting in the decision to make a tight joint without sacrificing the tool’s toughness. The three implements that are crafted from deer scapulae tell different stories (see below).

### Edge Morphologies, Raw Material Choices, and Tool Functions

#### *Si* Implements

Of the 50 identified *si* implements, 33 appeared to have also been used for other purposes. Nineteen of the 33 pieces show wear from processing plant and/or animal fibers (Table 6).

The edges of most scapular *si* are located at the medial ends of the bones. Two exceptions retain edges on the sides. Both artifacts were re-purposed, with the original medial edges for earth-working partially remaining on one sample, but completely missing on the other. The broken profiles on the sides were used. Results of use-wear analysis show that they were most likely used to process plant fibers.

**Fig. 16:**
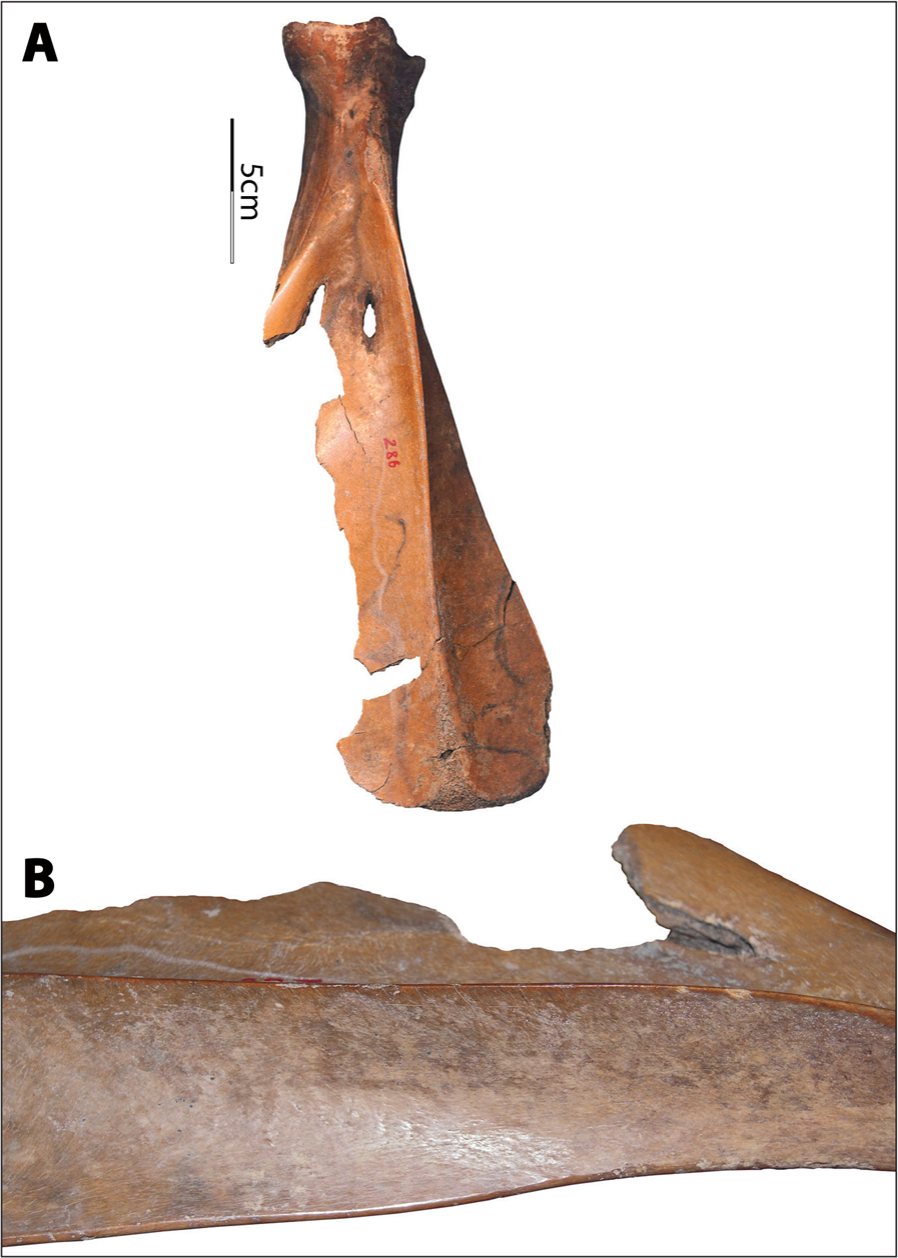
Example of a scapular fiber processer. **a** HMD 286. **b** Closer view of the spine of HMD 286. Note that a large portion of the spine was retained, intentionally sharpened, and used for additional abrasion in processing fiber

Most implements that were used primarily or exclusively for earth-working exhibit high degrees of spine removal. The implements that display fiber wear alone retain larger portions of their spines (Table 11). Sometimes, the spine itself is sharpened and used to provide additional abrasion in processing fiber (Fig. 16). Although a few fiber processors also show high degrees of spine removal, these implements may have originally been designed for earth-working (see below).

**Table 11:** Functions and degrees of spine removal

Worked material	III	IV	V	Total
Soil only	4	9	14	27
Both soil and fiber	5	9	6	20
Fiber only	8	15	2	25
Total	17	33	22	72

Degrees of spine removal: *III* moderate, *IV* almost completely removed, *V* completely flattened

**Fig. 17:**
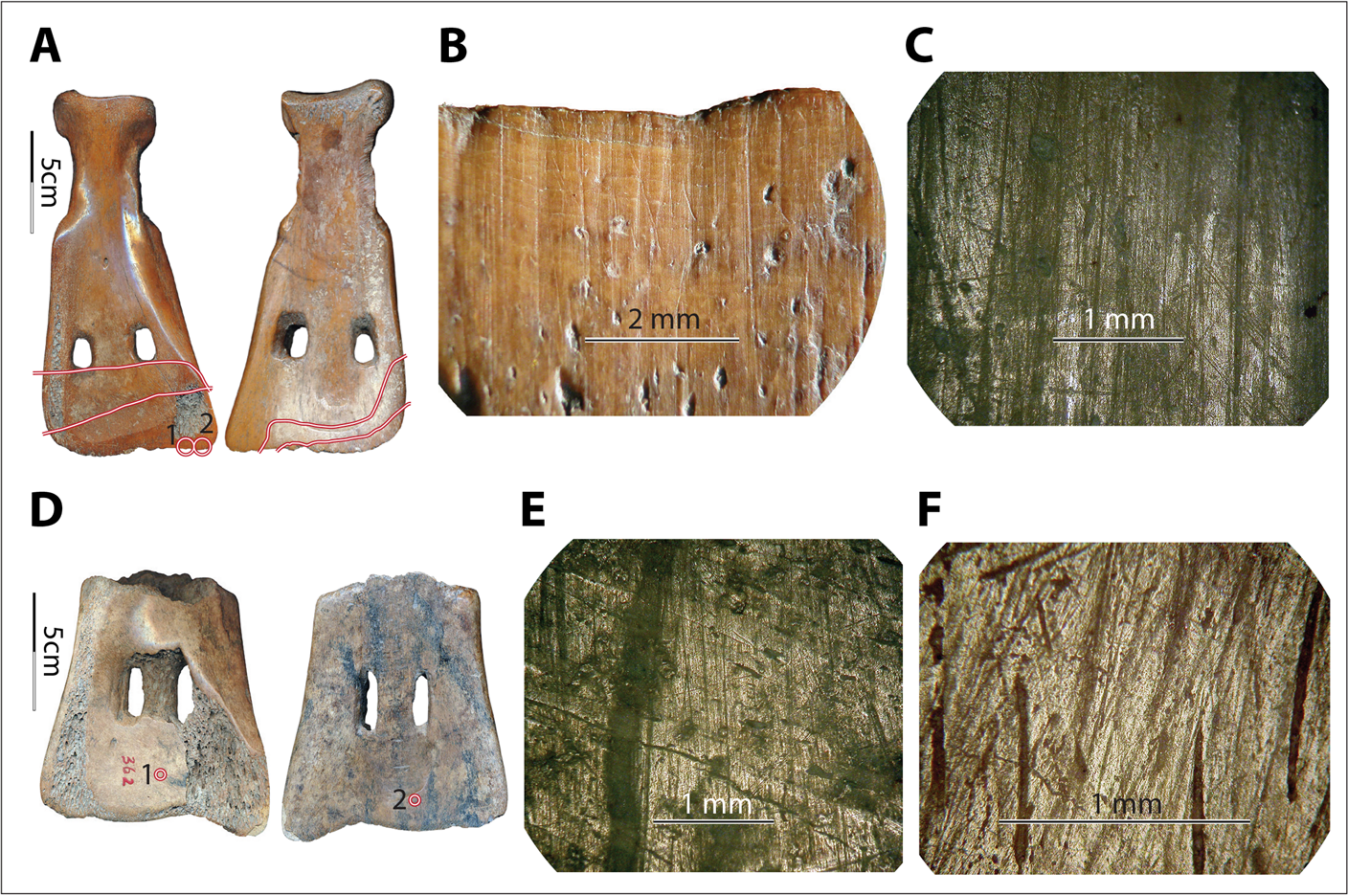
Two E-edged examples from Hemudu showing evidence of their use as earth-working implements. **a** Dorsal and ventral views of HMD 271 (the *lines* close to the edges indicate the extension of soil-derived striations visible to unaided eyes, while *lines* further off the edge indicate striations visible under a stereomicroscope). **b** Various sizes and lengths of soil-derived striations and scars (×20) at spot 1 of HMD 271. **c** Soil-derived wear (×50) at spot 2 of HMD 271. **d** Dorsal and ventral views of HMD 362. **e** Soil-derived wear (×50) at spot 1 of HMD 362. **f** Soil-derived wear (×100) at spot 2 of HMD 362

Nineteen implements appear to be E-edged. These implements are almost exclusively crafted from water buffalo scapulae, with only one sourced from a bear scapula. The edges of 17 out of the 19 implements show clear soil wear; 11 of them reflected contact with soil only. The two exceptions exhibit clear plant wear. However, three clues suggest that these two implements might originally have been used for earth-working: (1) the presence of ambiguous wear that may or may not have been soil-derived, (2) the presence of sophisticated hafting modifications, and (3) the complete removal and flattening of spines (Fig. 17).

**Fig. 18:**
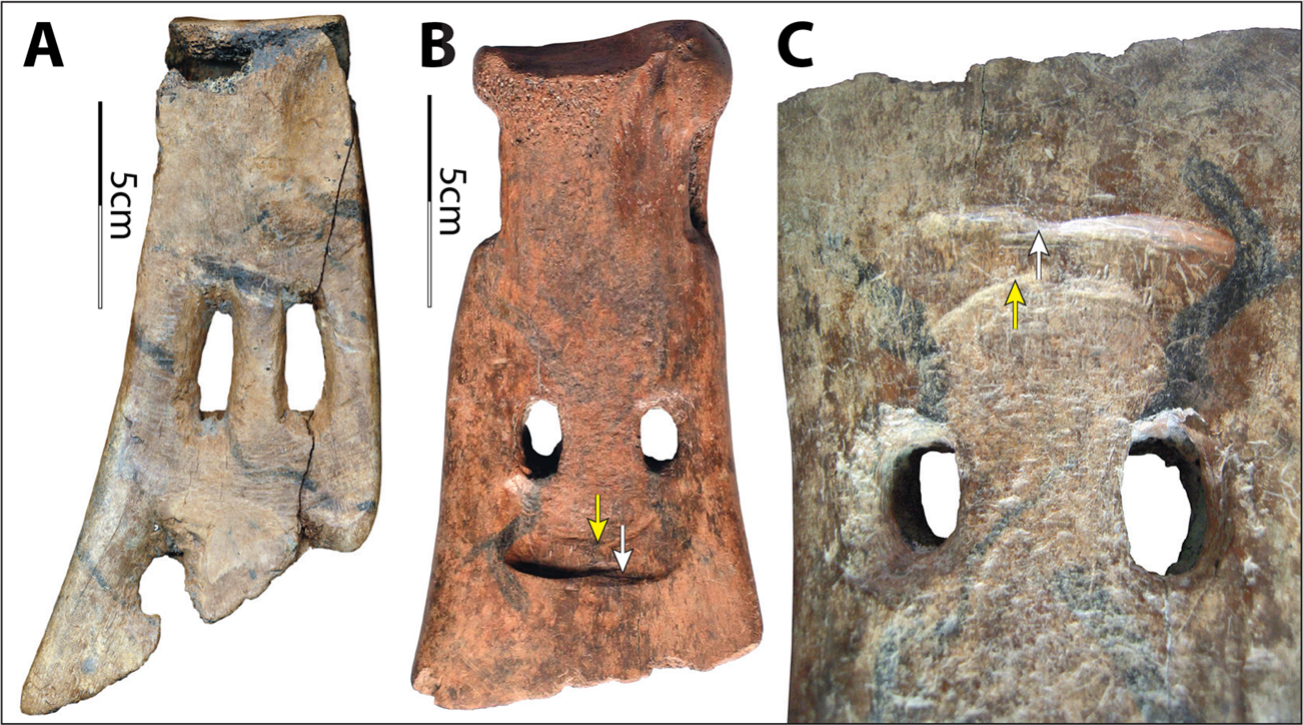
Examples from Tianluoshan showing readjustments of the implements’ hafting elements to maximize their residual use-lives. **a** TLST206(5):1 with two sets of perforations. **b** and **c** TLST203(3)A:1 showing two hafting grooves (subpanel **c** is a closer view of the end of the grooves; the *white arrowhead points* to the end of the longer groove while the *yellow arrowhead points* to the shorter one)

Perhaps because water buffalo scapulae were in short supply (Xie 2014, pp. 200–207), the E-edged implements were typically used until their lengths were reduced to non-functional dimensions. As the blades wore down and became shorter, the implements’ hafting elements were sometimes readjusted to maximize their residual use-lives. For example, the length of the hafting groove might be shortened or additional sockets or perforations crafted closer to the articulation the bone, leaving duplicated modifications as remnant traces on the implements (Fig. 18). Broken implements with significant residual use-lives continued to be used without substantial remodification for earth-working if the remaining morphology allowed or else recycled for other purposes including fiber processing. Alternatively, portions might be cut off broken implements, reshaped, and used for a variety of purposes.

#### Fiber Processors

**Fig. 19:**
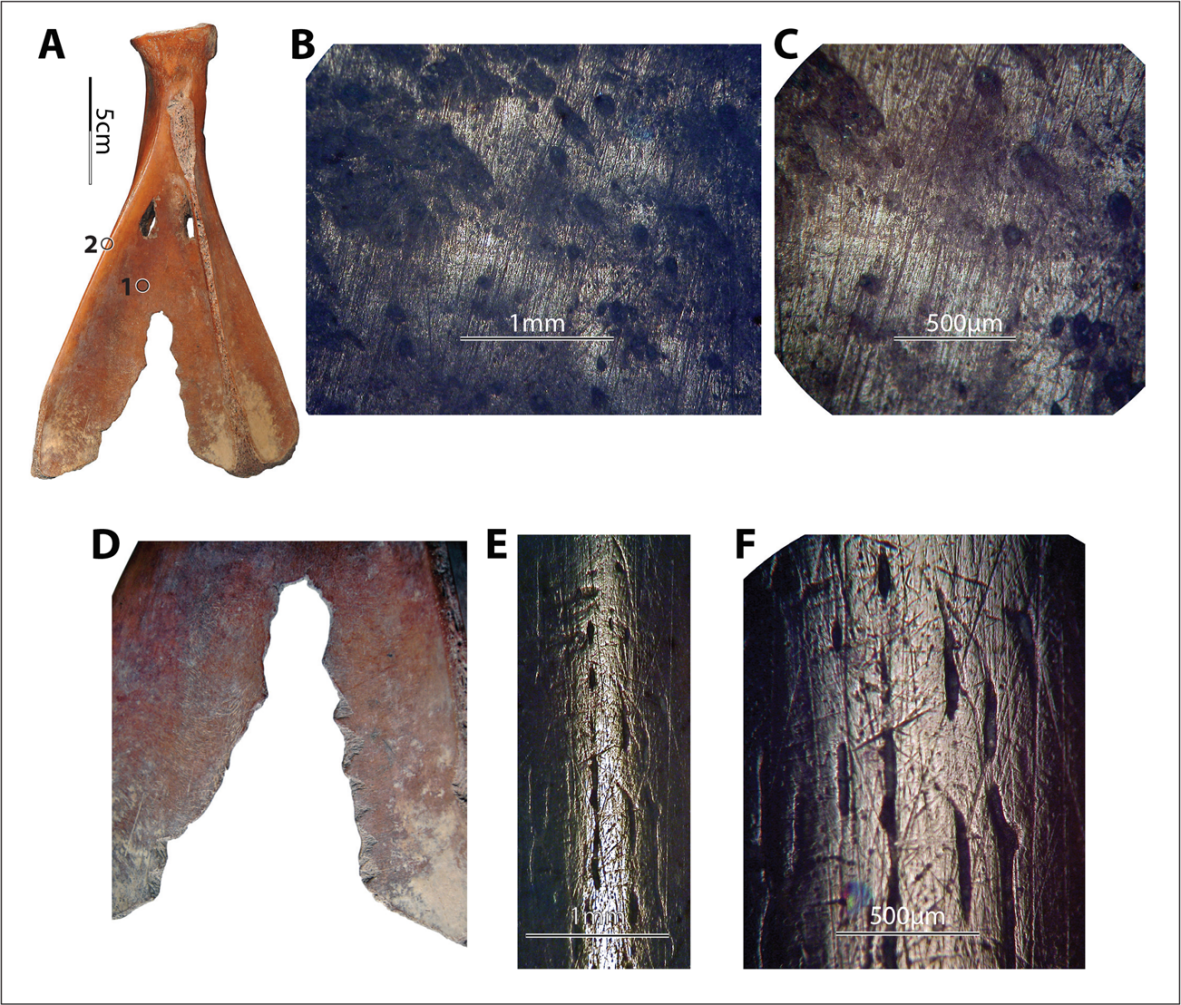
Evidence that HMD 336 was used as an earth-moving implement, which resulted in edge breakage, and then reused as a hide processor. **a** HMD 336. **b** Soil-derived wear (×50) at spot 1. **c** Soil-derived wear (×100) at spot 1. **d** A closer view of the two prongs of HMD 336. **e** and **f** Hide-derived wear (×50, ×100) at spot 2

Seven V-edged implements, all of them crafted from deer scapula, are identified with worked materials (Table 12). Such implements might have been used mainly as fiber processors, as ethnographic data suggest (Hofman 1980). One (HMD 336) of the seven implements was originally used for moving loosened soils in rice fields (with soil wear resembling experimental sample no. 6020); however, its remaining unsharpened edge indicates it was not produced for the explicit purpose of penetrating earth. This interpretation is consistent with the experimental results, which demonstrated that V-edged implements were inefficient at penetrating even soft agricultural soils. The traces on one side of the two prongs suggest that the prongs might have been formed by breakage from earth-working (Fig. 19).

**Table 12:** Morphologies and hafting designs of two-pronged scapular implements

Site	Species	Soil wear	Other function?	Perforations?	Neck modification	Groove?	Spine removal	Remodification	Prong length (cm)	Distance of prongs (cm)	Weight (g)	Length (cm)
HMD	Deer	y	Hide or plant	y	B	n	IV–V	n	7.8	8.8	109.5	20.4
HMD	Deer	n	Hide	y	A	y	III	n	6.9	9.7	109.9^a^	21.2^a^
HMD	Deer	n	Plant?	y	A	n	III	n	9.7	Missing	175.9^a^	22.8
HMD	Deer	n	Plant?	n	A	y	III	n	13.2	7.9	160^a^	25.4
HMD	Deer	n	Plant	y	C	y	IV	y	11.7^a^	Missing	159.8^a^	23.9
HMD	Deer	Undet	Plant or hide	y	A	y	III	n	6.1^a^	Missing	163.3^a^	19.7
TLS	Deer	Undet	Plant	n	B	y	IV	y	>6.6	5.4	191.8^a^	21.2

Neck modification: *A* none, *B* non-scored notches on the sides, *C* scored notches on the sides; spine removal: *III* moderate, *IV* almost completely removed, *V* completely flattened

^a^Means the implements or portions are incomplete

Hafting modifications of the V-edged deer scapular implements are usually minimal. Only one of the seven implements has hafting modifications comparable to those found on earth-working implements crafted from water buffalo bones. Such modifications of deer scapula are likely to result in breakage occurring along one of the perforations, taking one prong off of the V edge. Polish and rounding appearing on the broken profile suggest that the implement was used even after it was broken. Nothing indicates that the tool’s function changed after it was broken, although the manner of its use might have been adjusted accordingly. The hafting modifications appearing on the remaining six implements are much more restricted and with only a small portion of spine being removed (Tables 11 and 12).

#### Tool Functions and Edge Locations/Morphologies

**Fig. 20:**
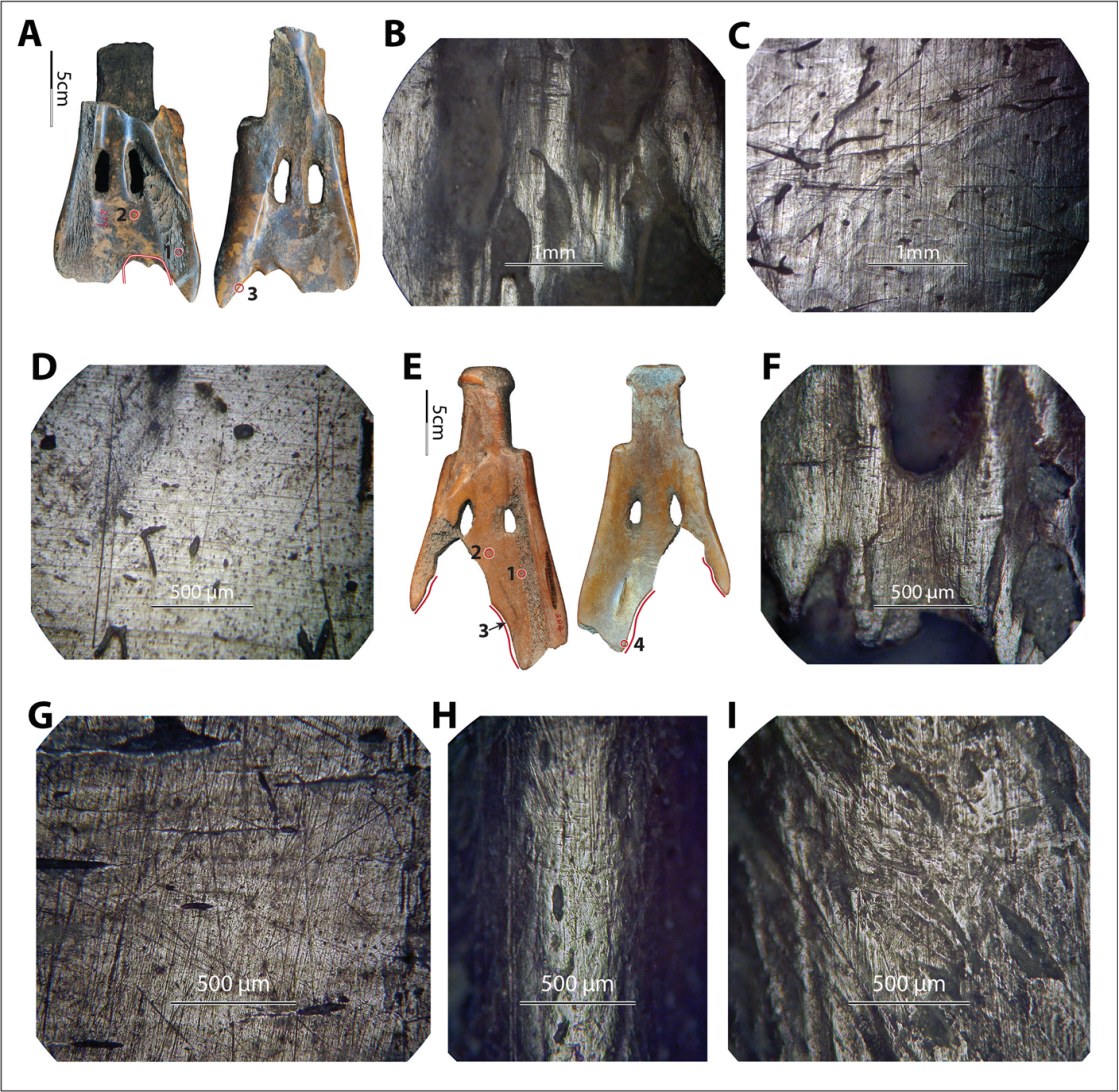
Two EV-edged examples showing evidence of their primary use as earth-working implements and secondary use as fiber processors. **a** Dorsal and ventral views of HMD 279 (the *line* indicates the extension of soil-derived striations visible to unaided eyes). **b** Soil-derived wear (×50) at spot 1 of HMD 279. **c** Soil-derived wear (×50) at spot 2 of HMD 279. **d** Plant-derived wear (×100) at spot 3 of HMD 279. **e** Dorsal and ventral views of HMD 304 (*lines* indicate the distribution of rounded edges produced by use). **f** Soil-derived wear (×100) at spot 1 of HMD 304. **g** Soil-derived wear (×100) at spot 2 of HMD 304. **h** Hide-derived wear (×100) at spot 3 of HMD 304. **i** Hide-derived polish covering soil-derived wear (×100) at spot 4 of HMD 304

Among the scapular implements with at least partial edges retained, 58 exhibit identified use-wear (Table 13), 27 of which have a combination of V and E edges (*i.e.*, EV-edged). Sixteen of the 27 EV-edged implements (1) show soil-like wear, (2) are crafted exclusively from water buffalo, (3) bear indications of sophisticated hafting modifications, and (4) have had their spines thoroughly removed. Use-wear analyses indicate that ten were originally employed in earth-working and reused for fiber processing. It is likely that the remaining EV-edged samples follow the same use and reuse pattern but the soil wear resulting from the tools’ original use is completely obscured. The two prongs on the EV-edged implements that exhibit soil wear differ morphologically from the prongs on the V-edged samples used exclusively for fiber processing, with the former showing shorter prongs, *i.e.*, shallower notches between the two prongs. These EV-edged samples may have originally been designed as E-edged implements and used for earth-working, which caused damage giving them the appearance of a V edge and were subsequently used to process fibers (Fig. 20).

**Table 13:** Functions and edge locations/morphologies

Worked material	E	ES	EV	S	V	Total
Soil only	11	0	6	0	0	17
Both soil and fiber	6	1	10	1	1	19
Fiber only	2	3	11	0	6	22
Total	19	4	27	1	7	58

Edge locations/morphologies: *E* straight edge at the medial end of the bone, *S* at the side of the bone, edge may or may not be straight, *V* two-pronged edge, *ES* coexistence of E and S edges, *EV* coexistence of E and V edges

#### Tool Functions and Raw Material Choices

Although the majority of the scapular *si* appear to have been crafted from water buffalo scapulae, it is not clear whether all implements made from water buffalo scapulae were designed and/or once used primarily for earth-working. Thirty-eight of the water buffalo scapular tools do not exhibit soil-like wear. Ten of them show fiber-like wear from processing plants and/or hides (Table 10), and the remaining 28 implements’ functions are unknown because of missing edges, weathering, and/or the presence of unidentified wear.

## Discussion

Our immediate research purpose was to identify the functions of the Hemudu scapular implements in order to evaluate the hypothesized Hemudu *si* agriculture (*i.e.*, an advanced Neolithic farming technique involving tillage).

### Summary of the Results

The results of our use-wear analyses, along with experimental and ethnographic evidence, suggest that some but not all of the Hemudu scapular implements might be designated as *si* (*i.e.*, tools for breaking ground and turning soils over to assist in seeding). Simply put, those crafted from water buffalo scapulae, especially those exhibiting a straight edge on the medial end, are most likely having been used as *si*. The ones crafted from scapulae of deer and/or other animals might have been designed and/or used for other purposes such as processing fibers .

Water buffalo scapulae, although in short supply, were preferred for crafting the scapular *si* implements, with a few examples having been made from deer scapulae for light tasks such as relocating soil. The implements were typically used, adjusted, and reused until they were reduced to non-functional dimensions. Broken *si* that are no longer suitable for earth-working tasks but with significant residual use-lives would have been recycled for other purposes, including fiber processing.

Results of quantitative measurements of soil physical properties and mathematical modeling suggest that the Hemudu scapular *si* are effective only when dealing with soft soils with PR values lower than 8 kg/cm^2^, with best working efficiency in soils with PR values around 4 kg/cm^2^ and below. In the Hemudu culture, the soils in which the scapular *si* could be effective and efficient would have included the rice fields and habitation areas on the margins of the wetlands where soils were very moist. Even in these soils, the use-lives of the scapular *si* might have been relatively short, resulting in high consumption of the tools to complete tillage in large areas of the rice fields at Tianluoshan.

Results of use-wear analysis suggest that the scapular *si* were used most commonly in agricultural contexts, but they were also occasionally used in construction contexts; in those contexts, they might have been used for lighter tasks, including relocating loose soil and breaking ground where the soil was moist and soft. Because of the challenges of tilling large rice fields without interruption and of penetrating ground in construction contexts, sophisticated modifications were created to ensure tight handle fastening on the scapular *si* implements. The tradeoff of the safe hafting designs affected the toughness of the overall implements, resulting in specific breakage patterns of the tools.

The implements made from deer scapulae, which were less modified than the water buffalo scapulae, show greater flexibility of morphological design and reflect a broader range of uses. Although use-wear traces on many deer scapular implements remain unidentified, one of the most common uses for these implements seems to have been processing fiber. The V edges were likely created to process fibers, although implements intended for other uses could have been re-purposed to this use if their broken edges happened to become V-edged.

### Implications of the Results in Relation to Hemudu Rice Cultivation

Only 50 out of 155 examined scapular implements have been identified as *si* implements. Even if all implements crafted from water buffalo scapulae that were examined, 78 pieces in total, were once used as *si* implements, that means half of the total number of examined implements did not function as *si*. Given that the *si* implements were uncovered from more than 3000 m^2^ of excavation areas and from anthropogenic deposits dating to a period spanning about a thousand years (7000–6000 BP) (Xie 2014: Table 2.3), *si* implements may have been used less abundantly or frequently in the Hemudu communities than we would expect from a society where people regularly practiced rice cultivation.

In addition, scapular *si* implements may not have functioned well in relatively hard soils, and their use-lives may have been rather short even in soft soils with little sand fraction. Consequently, the scapular *si* tradition in the Hemudu culture might have limited the people’s decisions on where and how much area to cultivate for rice, even given the natural wetland conditions. These technical constraints of the scapular *si* may partially explain why rice cultivation never became a mainstay in the Hemudu culture (Xie et al. 2015).

One may argue that the presence of abundant food resources in the area where the Hemudu culture sites are located allowed people to deploy their subsistence strategies more flexibly (Pan 2011; Zheng et al. 2012) and therefore could explain the delayed development of intensified rice cultivation. However, people living in many other places with similar food resources used different earth-working implements and developed a farming-dominated subsistence strategy sooner (Xie 2014). We believe that technological constraints, especially the kind of earth-working implements traditionally employed, played a role in shaping the subsistence strategies, not only on a technical level but, more importantly, in shaping strategic thinking regarding land use. We hope that future research on subsistence strategies will pay more attention to the technological aspects.

### Rethinking the Research Methods

As previously mentioned, the functional interpretations of archaeological scapular implements have been based on analogy, mostly morphological analogy, to ethnographic and historical implements. Although experiments have been conducted to test the hypothesized functions, those experiments were limited. These analogies have led to inconclusive results.

Reliable analogy requires that the two analogs share as many similarities as possible, including not only morphological similarities of the implements themselves but also similarities in their use contexts. We conducted our research with emphases on both aspects, and the results provided a base for re-evaluating previous research as well as building standardized database with scientific value for future research.

#### Evaluation on Different Literature Sources for Tool Functional Studies

Our experimental results clearly showed that the functionality of earth-working implements and the soil-derived wear they incur are both sensitive to soils’ physical properties.

The images of the V-edged implements appearing in the ancient pictorial stone with its agricultural theme and the wooden implements of similar morphology frequently found in Chinese Iron Age construction contexts provide inadequate evidence to conclude that these tools were used as earth-working implements. In an agricultural or construction context, a variety of tools in addition to earth-working implements would have been needed to fulfill purposes such as seeding and rearranging loose materials. Therefore, simple association with a behavioral context requiring earth-working is insufficient to establish the exact function of the implements. Even if the tools were indeed used for ground penetration in the contexts mentioned above, one should be cautious when drawing general conclusions concerning the functions of morphologically similar tools from other areas. The archaeological clues to V-edged tools’ function(s) come from northern or central China while the V-edged scapular implements come from the Hemudu culture on the Ningshao Plain in eastern China, where soil composition and physical properties are different. Our experimental results show that (scapular) tools that penetrated one ground sufficiently may not do so in another field. In addition, the natural morphology of scapulae imposes constraints on tool morphology, *i.e.*, the two prongs of the edge cannot be completely symmetrical because the scapular medial end is curved and uneven, while materials such as wood are free of this problem. This morphological difference may have kept the scapular V-edged implements from functioning the same way as V-edged implements crafted from alternative materials.

The implications of the ethnographic descriptions and previous experimental results were also restricted by the specific use contexts and morphologies of the subjects. For example, the scapular hoes used by American Indians on the Northern Plains were recorded as farming tools working in fine (alluvial) soils along the Missouri river (Henry 1988). However, as Wilson, noted in the early twentieth century, the scapular hoes were used with a cutting motion rather than a scraping motion, as is usual with iron hoes (Gilman and Schneider 1987, p. 32). Because our experimental results show that scapular implements could not effectively penetrate grounds even in relatively soft soils (*e.g.*, in field II with a PR value of 2.3 kg/cm^2^), we suspect that the rather awkward use motion of the scapular hoes by American Indians perhaps reflects the weak impact the tools could apply even when penetrating relatively soft, alluvial soils.

Interestingly, among numerous ethnographic accounts that recorded the American Indians scapular hoes, these tools are always mentioned as effective horticultural or farming implements; only Wilson noticed and mentioned the awkward use motion of scapular hoes. Further research is needed before we can conclude that scapular hoes did not function exactly as most ethnographic accounts suggest. Nevertheless, our experimental results, along with Wilson’s notes, remind us that ethnographic descriptions should be evaluated with caution, because their reliability is restricted by the visitor/recorder’s knowledge, interests, and biases, which will influence what is emphasized in his/her account.

Likewise, experimental results are reliable and useful only under specific situations. Davis’s positive comments on scapular implements were based on digging experiments conducted in fine, silty soil with replicated scapular implements crafted from cattle bones (Davis 1965). In contrast, Evans and Limbrey’s negative impressions of scapular tools were based on brief tests with unmodified ox and horse scapulae used in cemented sand with thin bands of pipe clay (Evans and Limbrey 1974, pp. 199–200). Based on our experimental results, we now know that these results are complementary. With quantitative measurements of soils’ physical properties and quantitative calculation, we conclude that scapular shovels are effective earth-penetrating tools only when dealing with soils with PR values lower than 8 kg/cm^2^, with best working efficiency in soils with PR values around 4 kg/cm^2^ and below. The fields where Davis conducted his research may have fallen within this PR range while those of Evans and Limbrey’s experiments most likely did not. These evaluations demonstrate that quantitative measurements of soil properties allow researchers to build additive databases where each work’s contribution will be of scientific value, even if done in isolation and in different soil contexts.

Our experimental results also suggest that close morphological emulation is critical for experimental evaluation of functionality, as scapular implements with adequate removal of the medial ends outperformed those without. This may also be one of the reasons why the unhafted, unmodifed scapulae employed by Evans and Limbrey (1974) were barely functional as scrapers in loosened soils.

Differences in tool morphology, as well as soil properties, can also explain why the scapular shovels used by the Jingpo people in southwest China were too fragile to penetrate the ground there, while those used in some of our experimental fields were functional.

#### Use-Wear Analyses and Associated Experimental Designs

Limited experimental data for each category of tool function have been an issue for many use-wear analysis research projects. Certain contact materials, such as hides and silica-rich plants, are commonly used in such experiments, and so datasets of use-wear patterns have accumulated over time, allowing cross-experimental comparison to achieve relatively reliable identifications. However, data on use-wear derived from soil are underrepresented in the dataset, restricting our ability to recognize earth-working tools; such recognition is crucial for the study of agricultural land use in the past. Our experiments significantly increase the dataset for soil-derived use-wear patterns. With quantitative description of the worked materials, our dataset is also of scientific value for future use and evaluation.

With reference to ethnographic and historical ethnographic accounts, and experimenting in the soils that prehistoric people might have contended with, our experimental data not only help to identify the scapular *si* but also allow further identification of their particular working contexts, either as agricultural tools, non-agricultural tools, or both. A combination of a low-power stereomicroscope and a high-power metallurgical microscope for use-wear analysis allowed us to scrutinize relatively large numbers of both experimental and archaeological samples and thereby strengthened the reliability of our use-wear identification on an assemblage level. Future additional image analysis will be helpful in distinguishing finer differences in use-wear across fields and/or soil types.

## Conclusions

With reference to experimental and ethnographic evidence and results of use-wear analysis, we conclude that *si* implements were present in the first millennium of the Hemudu culture, 7000–6000 BP. The majority of the *si* implements were crafted from water buffalo scapulae. However, the frequency with which the *si* implements appear seems to have been rather low and therefore it is impossible to conclude that they were employed on a regular basis. This finding supports the most recent recognition of the Hemudu subsistence strategy as a broad-spectrum diet complementary with low-level food production.

Because the scapular *si* implements could effectively function, though only under specific soil conditions, in the area where the Hemudu culture is situated, the long-term employment of these implements could have played a role in shaping people’s strategic thinking about agricultural land use. We propose that technological restriction provides an additional perspective for understanding the pathways to plant agriculture and future research should look more into this aspect.

## Footnotes

1 Shovels refer to the hafting methods with the blade affixed extending off the end of, with its axis in line with the handle.

2 A hoe is affixed perpendicularly to the handle with its cutting edge being at a right angle to the shaft.

3 The microscopes were purchased with the first author’s dissertation fellowship. Because microscopes produced by any well-recognized company were unaffordable, we purchased microscopes assembled by technicians from Beijing Crown Puca Technology Co., LTD, using components produced in China. To accommodate large samples, the metallurgical microscope used a large stand similar to the SZ-STL large stand associated with the Olympus BXFM upright metallurgical microscope. The stereo- and metallurgical microscopes shared one stand, so we could use only one of the two microscopes at a time. The microscopes, especially the metallurgical microscope, had various problems, but we managed to get the work done by investing much more time and labor into use-wear examination and recording. For example, because the observation tube was very heavy, when it was positioned in the cheaply made holder mount, the latter consistently move downwards. Even though the movement did not significantly affect use-wear observation, it made use-wear photographing extremely challenging. To produce one or two photos of publication quality, we often had to take hundreds of shots. We also had to take extremely detailed notes rather than relying on the photos to record use-wear observations. By sharing this experience, we want to make two points: (1) poor research conditions should not be an obstacle to achieving research goals; however, (2) ideal research conditions can significantly promote productivity and therefore should be pursued whenever possible.

4 One of our reviewers commented on having many times witnessed modern Chinese farmers using their hands to spit polish their tool handles and to wipe the tool clean. We too often witnessed similar behavior by modern Chinese farmers, who sometimes also pick up whatever materials are readily available on the ground, such as bunches of grass or straw, to clean their tools upon completion of their field work. All these behaviors could result in macro polish all over the tool surface. Future experiments and analyses are needed to evaluate whether the Hemudu people performed similar behaviors.
